# Discovery of Novel Bacterial Chalcone Isomerases by a Sequence‐Structure‐Function‐Evolution Strategy for Enzymatic Synthesis of (*S*)‐Flavanones

**DOI:** 10.1002/anie.202107182

**Published:** 2021-06-30

**Authors:** Hannes Meinert, Dong Yi, Bastian Zirpel, Eva Schuiten, Torsten Geißler, Egon Gross, Stephan I. Brückner, Beate Hartmann, Carsten Röttger, Jakob P. Ley, Uwe T. Bornscheuer

**Affiliations:** ^1^ Department of Biotechnology & Enzyme Catalysis Institute of Biochemistry Greifswald University Felix-Hausdorff-Strasse 4 17489 Greifswald Germany; ^2^ Symrise P.O. Box 1253 37603 Holzminden Germany

**Keywords:** biocatalysis, chalcone, chalcone isomerase, flavanone, flavonoid

## Abstract

Chalcone isomerase (CHI) is a key enzyme in the biosynthesis of flavonoids in plants. The first bacterial CHI (CHI_era_) was identified from Eubacterium ramulus, but its distribution, evolutionary source, substrate scope, and stereoselectivity are still unclear. Here, we describe the identification of 66 novel bacterial CHIs from Genbank using a novel Sequence‐Structure‐Function‐Evolution (SSFE) strategy. These novel bacterial CHIs show diversity in substrate specificity towards various hydroxylated and methoxylated chalcones. The mutagenesis of CHI_era_ according to the substrate binding models of these novel bacterial CHIs resulted in several variants with greatly improved activity towards these chalcones. Furthermore, the preparative scale conversion catalyzed by bacterial CHIs has been performed for five chalcones and revealed (*S*)‐selectivity with up to 96 % ee, which provides an alternative biocatalytic route for the synthesis of (*S*)‐flavanones in high yields.

## Graphical abstract

Novel bacterial chalcone isomerases and their variants identified by a Sequence‐Structure‐Function‐Evolution (SSFE) strategy are able to convert chalcones to flavanones with diverse substrate scopes and strict (*S*)‐stereopreference enabling a biocatalytic route to (*S*)‐flavanones.

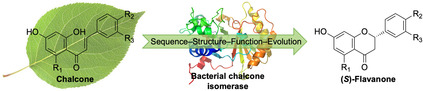

Nature produces an enormous variety of biomolecules, among which flavonoids are a large class of secondary metabolites mainly derived from plants. Flavonoids have many functions in plants, such as flower coloration, UV filtration, symbiotic nitrogen fixation, and floral pigmentation. In addition, they also act as chemical messengers, physiological regulators, cell cycle inhibitors, and antibiotics against plant diseases.^[1]^ In our long history of using medicinal herbs, there is substantial evidence that flavonoids show potential beneficial pharmacological properties for humans, such as anti‐infection, anti‐inflammatory, anti‐oxidant, anti‐tumor, anti‐aging, and immune response regulation.^[2]^ Recently, some flavonoids have been highlighted as potential medicine with anti‐coronavirus activity.^[3]^ Due to their various potential physiological benefits to humans, the study of the biosynthesis of flavonoids has been a research hotspot for several decades.^[4]^ The synthesis of flavonoids in plants is derived from the phenylpropanoid pathway (Scheme 1 a).^[4]^ Chalcone isomerase (CHI) cyclizes chalcones to form (*S*)‐naringenin which is one of the central intermediates for the diversification of flavonoids. As one of the key enzymes in the natural pathways of flavonoid biosynthesis, plant CHIs have been well studied^[5]^ with respect to their catalytic mechanism,^[6]^ protein structure,^[6b]^ classification,^[7]^ and evolution.^[8]^

**Scheme 1:**
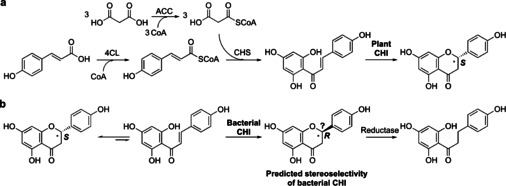
a) Biosynthesis^[4]^ and b) predicted biodegradation pathways^[9]^ of flavanones. 4CL: 4‐coumarate: CoA ligase, ACC: acetyl‐CoA carboxylase, CHS: chalcone synthase, CHI: chalcone isomerase, Reductase: enoate reductase or flavanone/flavanonol‐cleaving reductase, and CoA: coenzyme A.

The flavonoids contained in plants need to be degraded by microorganisms. Especially for some flavonoids with antibacterial activity, microorganisms have evolved biodegradation pathways to resist such flavonoids. Therefore, studying the pathway of microbial degradation of flavonoids can provide more clues about the biosynthesis and structural modification of flavonoids.^[10]^ The first bacterial CHI activity in *Eubacterium ramulus* for the anaerobic degradation of flavonoids was proposed by Schneider et al. in 2000.^[10a]^ The enzyme (CHI_era_) was then isolated from the cell‐free extracts of *E. ramulus*, which presented activity towards naringenin chalcone, isoliquiritigenin, and butein.^[11]^ After ten years without further reports, the gene of this bacterial CHI was identified from the genome of *E. ramulus* and cloned into *Escherichia coli* for recombinant expression in our group.^[12]^ Furthermore, we determined its crystal structure and suggested the reaction mechanism as reversible Michael addition catalyzed by His33 as a catalytic base abstracting the proton on C3, as well as its evolutionary uniqueness according to the homology search within protein databases.^[13]^ This bacterial CHI was then revealed to be a key enzyme catalyzing the conversion of the flavanone and flavanonol to the dihydrochalcone, together with an enoate reductase/flavanone‐ and flavanonol‐cleaving reductase (ERED/Fcr) (Scheme 1 b).[^9^, ^12^] Surprisingly, this is still the only bacterial CHI reported so far. However, the flavonoid structures are highly diverse due to the extensive hydroxylation, methylation, and glycosylation modifications on the C_6_‐C_3_‐C_6_ carbon framework. In addition to the huge amount of plant spoilage created every year, if the flavonoid degradation pathway involves a CHI as an important microbial degradation step, it is hard to imagine that microbial CHIs are rare. Besides, the substrate scope, stereoselectivity, evolution, and physiological role of bacterial CHIs in the degradation of flavonoids are also still unclear. These properties are not only important to understand the function and substrate scope of CHIs, but at the same time allow access to a toolbox of synthetically useful biocatalysts to make a range of important flavonoids relevant for the flavor market. Herein, we report the discovery of novel bacterial CHIs and their substrate specificity differentiation as well as stereoselectivity. We have designed a Sequence‐Structure‐Function‐Evolution (SSFE) strategy to identify novel and interesting candidate CHIs from protein databanks to expand the substrate scope of known CHIs for the biosynthesis of flavanones.

Gene mining based on a known protein sequence is an effective strategy for finding novel enzymes from gene or protein databases. Phylogenetic analysis allows us to determine highly probable evolutionary relations in datasets of protein or DNA sequences based on the maximum likelihood method.^[14]^ This can be used to identify sequences with potentially similar or related activities (SSFE, Sequence step). First, we searched the non‐redundant protein sequence database of Genbank with the protein sequence of CHI_era_ and the fragment sequences of its catalytic and solvent‐exposed domains^[13]^ as query to collect the protein sequences of bacterial CHI‐like proteins and further constructed a phylogenetic tree (Figure 1, a full version is included in Figure S2). The phylogenetic tree consists of two main branches, where most sequences are included in branch A (67 proteins), with sub‐branches 1–13, including the sub‐branch 7 (highlighted in black) with our starting sequence CHI_era_. The other branch (branch B) consists of an enzyme cluster with lower sequence similarity to CHI_era_. However, it is still possible that one may find related activities and sequences in this branch and this would shine light on the evolutionary origin of bacterial CHIs. The original host sources included in the dataset show that bacterial CHI‐like proteins are widespread, with some intestinal bacteria, such as *Clostridium*, *Eubacterium*, *Roseburia*, *Lachnoclostridium*, *Mogibacterium*, *Butyrivibrio*, and *Mobilibacterium*, but also some environmentally derived bacteria, including *Vibrio* and *Aminicella*, the thermophilic bacteria *Tepidanaerobacter*, and some acetogenic bacteria such as *Acetoanaerobium* and *Halophaga*. These bacteria include Gram‐negative and Gram‐positive strains, but are mostly anaerobic or facultatively anaerobic bacteria. This indicates that the metabolic pathway involved in bacterial CHIs might require an anaerobic environment.

**Figure 1:**
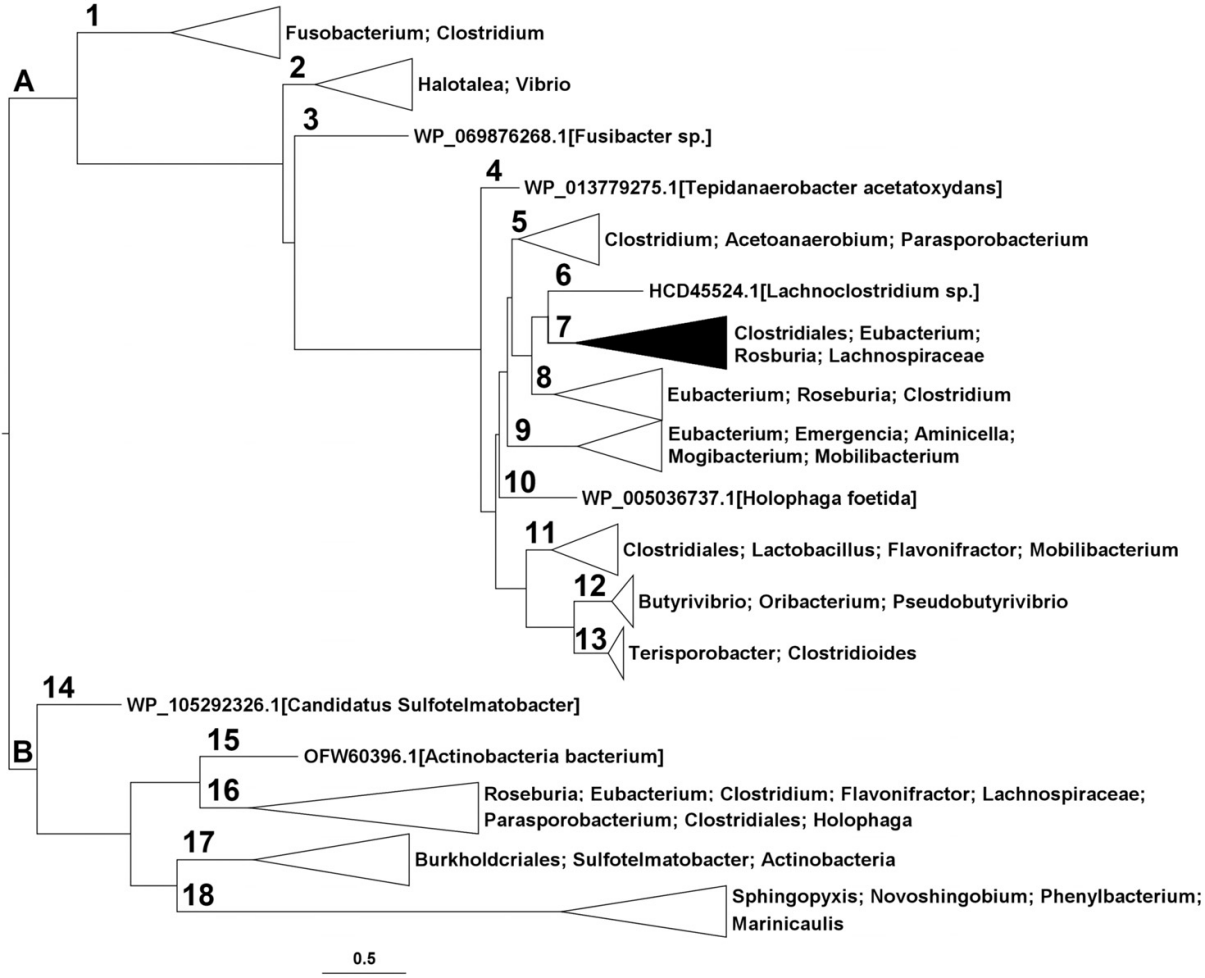
Phylogenetic tree of potential bacterial CHIs. A full version is included in Figure S2. The branch where CHI_era_ locates is highlighted in black.

In the phylogenetic analysis, the bacterial CHI‐like proteins are divided into clusters based on their sequence similarity. However, enzymatic properties can be predicted not only from homology of the sequences and residues in the catalytic site, but also from the substrate binding pockets and protein folding structures. These attributes are poorly reflected in the alignment of protein sequences due to the flexibility of peptide folding and our general lack of understanding of protein folding. Therefore, systematic analysis of the sequence and structural characteristics of proteins on each branch can effectively predict the activity and partial structure‐related enzymatic properties of these proteins (SSFE, Structure step). We submitted all 88 protein sequences of the phylogenetic tree to SWISS‐MODEL^[15]^ to model their three‐dimensional structures. The alignment of each simulated structure to the crystal structure of the CHI_era_ shows that all proteins in the phylogenetic tree have quite similar folds, especially the ones located on branch A. CHI_era_ contains two unique ferredoxin‐like domains; the catalytic domain and the solvent‐exposed domain.^[13]^ From the structure alignment (Figure 2 a), it is clear that the folding of proteins in branch A is conserved and consistent with CHI_era_. However, the catalytic domain and the solvent‐exposed domain are slightly different for the proteins in branch 14, as well as the branches 15 to 18. Extra residues between the α‐helix (α6) and β‐sheet (β6)^[13]^ in the solvent‐exposed domain form loops with high flexibility (Figure 2 a, Loop1). The α‐helixes (α3, α4, and α5)^[13]^ in the catalytic domain are completely restructured as flexible loops (Figure 2 b, Loop2). Due to this significant difference in the catalytic domain structures to CHI_era_, we predicted that the proteins in branch B might have no CHI activity, which has been proved further by activity screening (data not shown). Therefore, further analysis mainly focused on the proteins in branch A.

**Figure 2:**
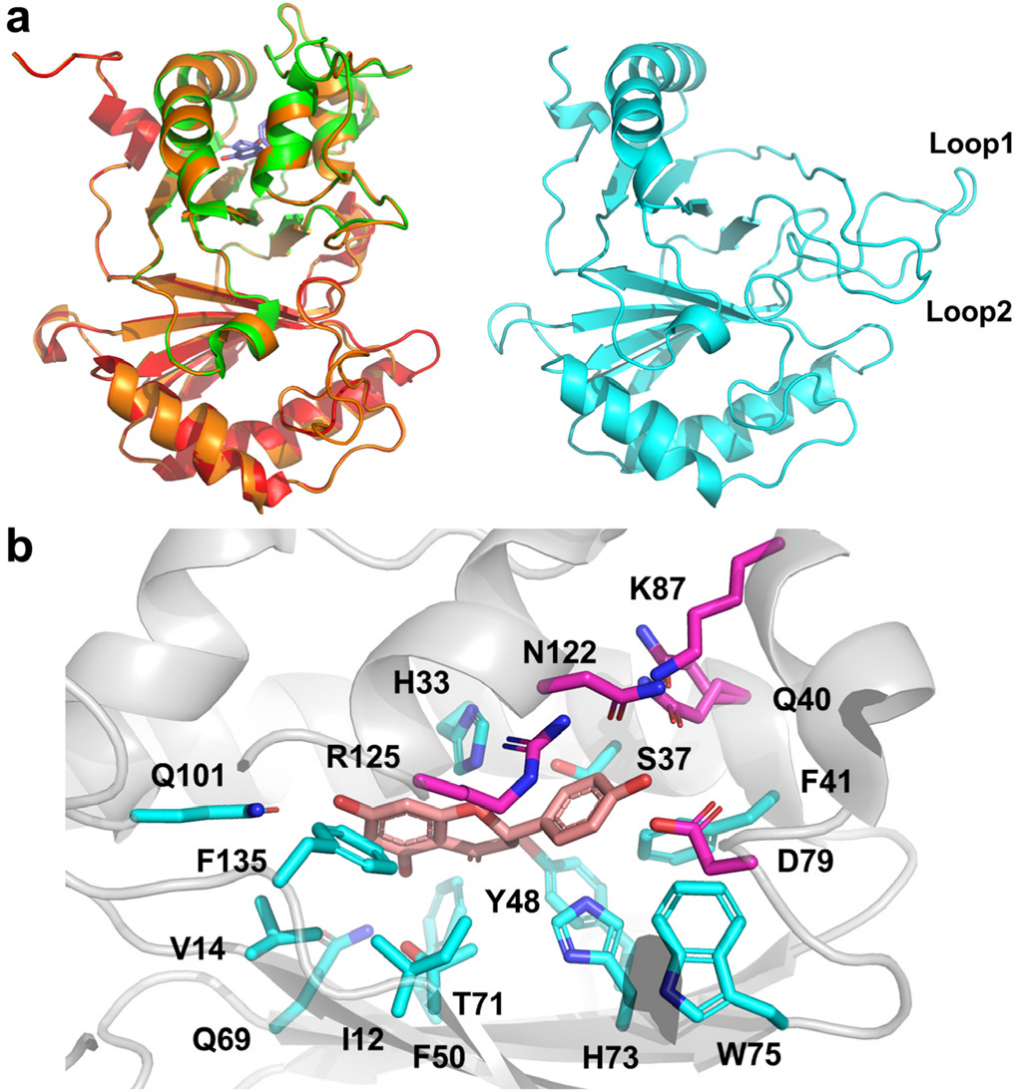
a) Examples of the overall structure analysis of potential bacterial CHIs. Green: catalytic domain of CHI_era_. Red: solvent‐exposed domain of CHI_era_. Blue: (*S*)‐naringenin. Orange: simulated protein structure of WP_077390608.1 from branch 11. Cyan: simulated protein structure of WP_105292327.1 from branch 17. b) Active pockets of CHI_era_. Orange: (*S*)‐naringenin. Magenta: mutagenesis sites of CHI_era_.

Next, we focused on the catalytic domains, particularly the active pockets, to assign potential activities and substrates to the sequences in each sub‐branch. According to the crystal structure of CHI_era_,^[13]^ the substrate binding pocket contains 21 residues (Figure 2 b), where Gln101 binds 7‐OH, Glu69 and Thr71 have co‐hydrogen binding to 6′‐OH in the chalcone, Arg125 probably stabilizes the 2′‐OH together with Tyr48 binding to 9=O, His33 is the key residue for Michael addition, and Asn122, Asp79, Lys87, and Gln40 are corresponding residues for 4‐OH binding. In addition, Phe135, Ile29, Val14, Phe50, and Ile12 are involved in the binding pocket for the A‐ring in the flavanone/chalcone, while Arg125, His73, Ser37, Glu91, Trp75, and Phe41 are related to the B‐ring binding. We made a list of residues in the active pockets of the unknown sequences corresponding to these catalytically relevant residues. The residues are described by numbers that refer to the respective amino acid positions in CHI_era_ to avoid confusion (Table S1). From this list, it becomes obvious that all proteins in branch A contain the key catalytic residue His33, which can be considered as a clear indication of CHI activity. Tyr48 is also highly conserved, which stabilizes 9=O.^[13]^ Moreover, the Gln69, Thr71, and Gln101 residues responsible for interactions with the A‐ring of the substrate are also highly conserved, except for the proteins in the branches 1 and 2 distantly located from CHI_era_ in branch A. Regarding to the relatively few structural modifications on the A‐ring of flavanones, such a conservative A‐ring binding pocket is in line with our expectations to fit the limited variety. In contrast, the binding and positioning of amino acids in the B‐ring binding are much less conserved. The most important differences are found in the binding amino acids Asn122, Asp79, Lys87, and Gln40. The variety of amino acids at these sites includes polar and non‐polar amino acids with long and short side chains, which seems to suggest that both hydroxy and methoxy modifications on the B‐ring of chalcones are accepted. As we speculated, there might exist more bacterial CHIs in nature adapted to the diversity of flavonoid structures. The diverse active pockets indicate that these potential bacterial CHIs very likely have different substrate specificities.

Several sequences containing representative substrate binding pockets from each branch (13 in total) were selected for gene synthesis and characterization (SSFE, Function step). All candidate proteins (wild‐type CHIs from phylogenetic analysis, all recombinantly expressed in *E. coli* and used as purified enzymes) were tested for the conversion of a range of chalcones with modifications in their A‐ and B‐rings, including naringenin chalcone (**1 a**), eriodictyol chalcone (**2 a**), homoeriodictyol chalcone (**3 a**), hesperetin chalcone (**4 a**) and 4‐*O*‐methylbutein (**5 a**) (Scheme 2). The results are shown in Figure 3 and Table S2. Among the 14 wild‐type bacterial CHIs (including CHI_era_), CHI_era_ presented surprisingly high activity towards **1 a** (270 U mg^−1^). CHI6 belongs to the same branch with the same active pocket as CHI_era_. However, its activity towards **1 a** is only 68.3 U mg^−1^, but 123.6 U mg^−1^ towards **2 a**. Therefore, their activity differences might be caused by the entire enzyme sequence and structure, rather than just a more suitable substrate binding model. **3 a** contains a *meta*‐methoxy moiety in the B‐ring, leading to an approx. 50 % decrease in activity for most wild‐type CHIs in comparison to **2 a**. CHI2 and CHI4 have relatively good activity towards **4 a** (93.5 U mg^−1^ and 84.5 U mg^−1^, respectively). Both have a proline (P79) instead of an aspartate (D79) as in CHI_era_. This residue causes less steric hindrance, allowing the active site to accept the *para*‐methoxy moiety present in the B‐ring. Compared to **4 a**, **5 a** does not contain a hydroxy moiety at C6′. Although the hydroxy group at C2′ can turn into a C6′ hydroxy group due to the rotation of the C1′−C9 bond, this is not the energy minimum structure of **5 a**. Therefore, the specific activity of most wild‐type CHIs towards **5 a** is less than 1 U mg^−1^ (Table S2). CHI7 shows the highest activity of up to 1.2 U mg^−1^. The activity screening of potential bacterial CHIs confirmed CHI activity for all candidate enzymes. Thus, it can be inferred that the proteins present in branch A are likely all CHIs (66 novel bacterial CHIs plus CHI_era_). The relatively low activity of some enzymes might be improved by fine‐tuning of the reaction conditions. For example, in order to improve the stability of chalcones in aqueous solution, the reaction was performed at alkaline conditions, pH 8, which might not be the optimal pH for bacterial CHIs.^[12]^ In addition, CHI1 is close to the root of the phylogenetic tree, but shows almost the same activity to various chalcones. Therefore, it can be speculated that the CHI activity was obtained at the root of the phylogenetic tree and has continued to differentiate into various substrate specificities.

**Figure 3:**
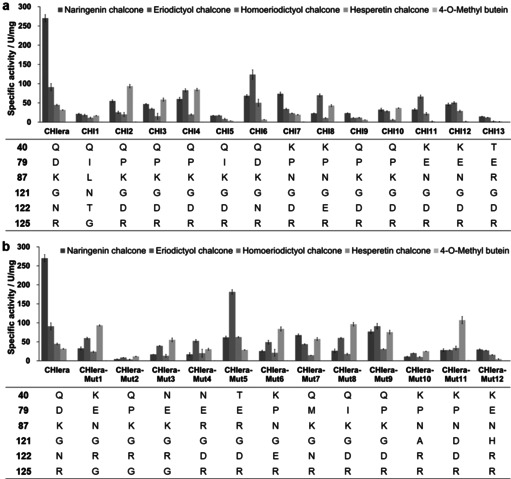
Substrate scope of a) bacterial CHIs and b) CHI_era_ mutants. The table of mutations shows variants in the substrate binding pocket of wild‐type CHIs and CHI_era_ mutants. The reaction was carried out with 100 μm chalcones in phosphate buffer (50 mm, pH 8.0).

**Scheme 2:**
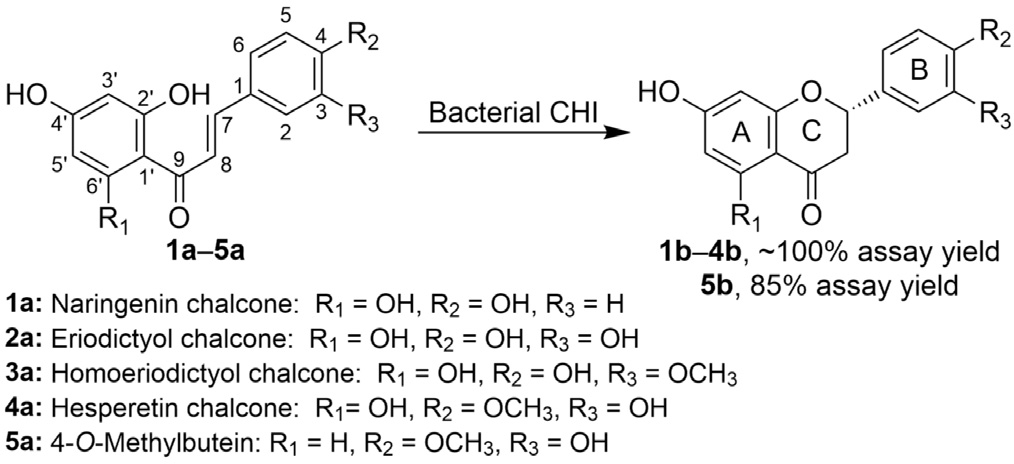
Bioconversion of chalcones to (*S*)‐flavanones by novel bacterial CHIs and CHI mutants.

The systematic analysis of the CHI sequences and structures and activity screening above provides useful hints about the natural evolution of active pockets for bacterial CHIs. We believe that this natural diversity is most likely a shortcut to guide the mutagenesis of bacterial CHIs leading to extended substrate scope (SSFE, Evolution step). We introduced mutations in the active pocket of CHI_era_ (12 variants in total) to cover most substrate pocket types as identified from the structure analysis and the phylogenetic tree. Among the 12 CHI_era_ variants, all lost the high activity towards **1 a** (Figure 3). However, the activity of CHI_era_Mut5 towards **2 a** (181.3 U mg^−1^) is doubled compared to the wild‐type and this variant also has improved activity towards **3 a** (62.2 U mg^−1^). From simulations of the active pocket of CHI_era_‐Mut5 with the substrate (Figure S4), it seems that K87R and D47E offer hydrogen bonding to the *para*‐hydroxy group and slightly shift the binding position of the B‐ring in the active pocket, which probably results in better binding of the *meta*‐hydroxy group to E91. Compared with the wild‐type CHI_era_, CHI_era_‐Mut11 shows greatly improved activity towards **4 a** (107.1 U mg^−1^), similar to CHI_era_‐Mut8 (96.4 U mg^−1^), CHI_era_‐Mut1 (93.2 U mg^−1^), CHI_era_‐Mut6 (83.9 U mg^−1^), and CHI_era_‐Mut9 (75.5 U mg^−1^). CHI_era_‐Mut6, 8, 9, and 11 contain either a proline or isoleucine at position 79, like CHI2, which has similar activity towards **4 a** (93.5 U mg^−1^). However, CHI_era_‐Mut1 and 3 have a D79E mutation which contains an even longer side chain. Alternatively, R125 in CHI_era_‐Mut1 and 3 is replaced by Gly to offer more room to adapt the *para*‐methoxy moiety of **4 a**. This provides a new substrate binding pocket for improving the activity of bacterial CHIs towards **4 a**. CHI_era_‐Mut11 has the highest activity towards **5 a** (1.4 U mg^−1^) of all variants. Although these CHI_era_ variants only represent some of the substrate binding pockets included in the phylogenetic tree, these results clearly show that the activity towards modified B‐ring chalcones can be improved greatly by semi‐rational design based on the results from Sequence‐Structure‐Function analysis. In some cases, an extensive collection of natural diversity can offer shortcuts to obtain suitable pockets for improved substrate binding.

Aside from the substrate scope of the novel bacterial CHIs, we investigated the stereoselectivity. Plant‐derived CHIs have been confirmed to have a strict (*S*)‐stereoselectivity.^[5]^ However, ERED/Fcr can directly reduce (*R*)‐naringenin, which indicates that bacterial CHIs are probably also able to isomerize (*S*)‐naringenin to the (*R*)‐isomer through its chalcone in aqueous solution and form a cascade reaction with ERED/Fcr to convert flavanones to dihydrochalcones.^[9]^ However, direct evidence of bacterial CHIs with (*R*)‐stereoselectivity is missing. Stereoselectivity was now determined using **1 a** as substrate and mainly (*S*)‐naringenin was detected in the reaction with a ratio of approx. 9:1 (*S*:*R*) for CHI_era_ and the novel identified bacterial CHIs (CHI1–CHI13, Figure S5a). Considering to the rapid self‐cyclization of chalcones in aqueous solution to generate racemic flavanones, this optical purity measurement proved that bacterial CHIs are (*S*)‐selective. Furthermore, we also determined in this work the stereoselectivity of the CHI and mutants with the highest activity (CHI_era_, CHI_era_‐Mut5, and CHI_era_‐Mut11) towards **1 a**–**5 a** (Figure S5b). These three enzymes presented relatively strict (*S*)‐stereoselectivity towards **1 a** and **4 a**, but almost no stereoselectivity for **2 a** and **3 a**, which shows the great influence of the structure and position of the substituents on the B‐ring on the stereoselectivity of the CHIs. CHI_era_‐Mut11 showed high stereoselectivity (9:1) towards 4‐*O*‐methylbutein. Although we could not identify whether it is (*R*)‐ or (*S*)‐stereopreference—due to the lack of standard compounds—we speculate that CHI_era_‐Mut11 is very likely to have (*S*)‐stereoselectivity for 4‐*O*‐methylbutein, referring to the same B‐ring structure of 4‐*O*‐methylbutein and hesperetin and their similar chiral‐HPLC results. These results show that the previously assumed degradation approach of (*S*)‐naringenin through chalcone, (*R*)‐naringenin, and dihydrochalcone in bacterial CHIs (Scheme 1 b)^[9]^ is most likely incorrect. Since the natural active flavanones are all in the (*S*)‐configuration, these bacterial‐derived CHIs with various substrate regioselectivities provide a diverse toolkit of enzymes to replace the plant CHIs for the heterologous synthesis of (*S*)‐flavanones, also keeping in mind that the bacterial CHIs are much easier to express recombinantly. Therefore, we have used CHI_era_, CHI_era_‐Mut5, and CHI_era_‐Mut11 as biocatalysts and achieved the enzymatic preparative scale synthesis of naringenin, eriodictyol, homoeriodictyol, hesperetin, and 7,3′‐dihydroxy‐4′‐methoxyflavanone with yields of 85–100 %, respectively.

In summary, we successfully identified novel bacterial CHI‐like proteins from Genbank using the SSFE strategy and confirmed them to have CHI activity. Several wild‐type CHIs as well as the designed variants show high activity towards several chalcones. Thus, the SSFE strategy has been demonstrated as a useful tool to mine and improve novel and useful candidate enzymes. Finally, we confirmed that bacterial CHIs have (*S*)‐stereopreference, which provides an alternative biocatalytic pathway for the synthesis of (*S*)‐flavanones with high yields.

## Conflict of interest

The authors declare no conflict of interest.

## Supporting information

As a service to our authors and readers, this journal provides supporting information supplied by the authors. Such materials are peer reviewed and may be re‐organized for online delivery, but are not copy‐edited or typeset. Technical support issues arising from supporting information (other than missing files) should be addressed to the authors.

Supplementary

## References

[1] M. L. F. Ferreyra, S. P. Rius, P. Casati, Front. Plant Sci. 2012, 3, 222. 23060891 10.3389/fpls.2012.00222 PMC3460232

[2] B. Romano, E. Pagano, V. Montanaro, A. L. Fortunato, N. Milic, F. Borrelli, Phytother. Res. 2013, 27, 1588–1596. 23824931 10.1002/ptr.5023

[3] T. Goris, A. Perez-Valero, I. Martinez, D. Yi, L. Fernandez-Calleja, D. San Leon, U. T. Bornscheuer, P. Magadan-Corpas, F. Lombo, J. Nogales, Microb. Biotechnol. 2021, 14, 94–110. 33047877 10.1111/1751-7915.13675 PMC7675739

[4] R. P. Pandey, P. Parajuli, M. A. G. Koffas, J. K. Sohng, Biotechnol. Adv. 2016, 34, 634–662. 26946281 10.1016/j.biotechadv.2016.02.012

[5] Y. C. Yin, X. D. Zhang, Z. Q. Gao, T. Hu, Y. Liu, Mol. Biotechnol. 2019, 61, 32–52. 30324542 10.1007/s12033-018-0130-3

[6a] J. M. Jez, J. P. Noel, J. Biol. Chem. 2002, 277, 1361–1369; 11698411 10.1074/jbc.M109224200

[6b] J. M. Jez, M. E. Bowman, R. A. Dixon, J. P. Noel, Nat. Struct. Biol. 2000, 7, 786–791. 10966651 10.1038/79025

[7a] L. Ralston, S. Subramanian, M. Matsuno, O. Yu, Plant Physiol. 2005, 137, 1375–1388; 15778463 10.1104/pp.104.054502 PMC1088328

[7b] N. Shimada, T. Aoki, S. Sato, Y. Nakamura, S. Tabata, S. Ayabe, Plant Physiol. 2003, 131, 941–951. 12644647 10.1104/pp.004820 PMC166860

[8a] M. Kaltenbach, J. R. Burke, M. Dindo, A. Pabis, F. S. Munsberg, A. Rabin, S. C. L. Kamerlin, J. P. Noel, D. S. Tawfik, Nat. Chem. Biol. 2018, 14, 548–555; 29686356 10.1038/s41589-018-0042-3

[8b] M. N. Ngaki, G. V. Louie, R. N. Philippe, G. Manning, F. Pojer, M. E. Bowman, L. Li, E. Larsen, E. S. Wurtele, J. P. Noel, Nature 2012, 485, 530–533. 22622584 10.1038/nature11009 PMC3880581

[9] A. Braune, M. Gutschow, M. Blaut, Appl. Environ. Microbiol. 2019, 85, e01233-19. 31375488 10.1128/AEM.01233-19 PMC6752008

[10a] H. Schneider, M. Blaut, Arch. Microbiol. 2000, 173, 71–75; 10648107 10.1007/s002030050010

[10b] M. Blaut, L. Schoefer, A. Braune, Int. J. Vitam. Nutr. Res. 2003, 73, 79–87. 12747214 10.1024/0300-9831.73.2.79

[11] C. Herles, A. Braune, M. Blaut, Arch. Microbiol. 2004, 181, 428–434. 15127184 10.1007/s00203-004-0676-2

[12] M. Gall, M. Thomsen, C. Peters, I. V. Pavlidis, P. Jonczyk, P. P. Grünert, S. Beutel, T. Scheper, E. Gross, M. Backes, T. Geißler, J. P. Ley, J. M. Hilmer, G. Krammer, G. J. Palm, W. Hinrichs, U. T. Bornscheuer, Angew. Chem. Int. Ed. 2014, 53, 1439–1442; 10.1002/anie.201306952 24459060

[13] M. Thomsen, A. Tuukkanen, J. Dickerhoff, G. J. Palm, H. Kratzat, D. I. Svergun, K. Weisz, U. T. Bornscheuer, W. Hinrichs, Acta Crystallogr. Sect. D 2015, 71, 907–917. 25849401 10.1107/S1399004715001935

[14] J. B. Mitchell, Curr. Opin. Struct. Biol. 2017, 47, 151–156. 29107208 10.1016/j.sbi.2017.10.004

[15] A. Waterhouse, M. Bertoni, S. Bienert, G. Studer, G. Tauriello, R. Gumienny, F. T. Heer, T. A. P. de Beer, C. Rempfer, L. Bordoli, R. Lepore, T. Schwede, Nucleic Acids Res. 2018, 46, W296–W303. 29788355 10.1093/nar/gky427 PMC6030848

