# Supplementary material for: Discovery of Novel Bacterial Chalcone Isomerases by a Sequence‐Structure‐Function‐Evolution Strategy for Enzymatic Synthesis of (S)‐Flavanones
Source: Angew Chem Int Ed Engl. 2021 Jun 30;60(31):16874–9. doi: 10.1002/anie.202107182 (PMC8361940; doi:10.1002/anie.202107182)
Supplement: Supplementary file 1 — Supplementary [file ANIE-60-16874-s001.pdf]

Angewandte Chemie
Eine Zeitschrift der Gesellschaft Deutscher Chemiker

Supporting Information

Discovery of Novel Bacterial Chalcone Isomerases by a Sequence-Structure-Function-Evolution Strategy for Enzymatic Synthesis of (*S*)-Flavanones

Hannes Meinert[+], Dong Yi[+], Bastian Zirpel, Eva Schuiten, Torsten Geißler, Egon Gross, Stephan I. Brückner, Beate Hartmann, Carsten Röttger, Jakob P. Ley,* and Uwe T. Bornscheuer**

anie_202107182_sm_miscellaneous_information.pdf

Table of Contents

Experimental Procedures 3
Materials 3
Protein sequence analysis and phylogenetic tree building 3
Protein sequence–structure analysis 3
Mutagenesis of CHI_{era} 3
Expression and purification of bacterial CHIs 3
Assay for bacterial CHIs 3
Stereoselectivity determination of CHI_{era} 3
NMR experiments 4
Enzymatic synthesis of naringenin (1b) by CHI_{era} 4
Enzymatic synthesis of eriodictyol (2b) by CHI_{era}Mut5 4
Enzymatic synthesis of homoeriodictyol (3b) by CHI_{era}Mut5 4
Enzymatic synthesis of hesperetin (4b) by CHI_{era}Mut11 4
Enzymatic synthesis of 7,3'-dihydroxy-4'-methoxyflavanone (5b) by CHI_{era}Mut11 5
Additional Results and Discussion 6
Figure S1. Full protein sequence alignment of potential bacterial CHIs 6
Figure S2. Full phylogenetic tree of potential bacterial CHIs 7
Figure S3. SDS-PAGE for expression and purification of potential bacterial CHIs 8
Figure S4. Simulation of the active site pocket of CHI_{era}Mut5 9
Figure S5. Stereoselectivity determination of bacterial CHIs 10
Figure S6. Optical purity determination of naringenin products 11
Figure S7. Optical purity determination of hesperetin products 12
Figure S8. Optical purity determination of eriodictyol products 13
Figure S9. Optical purity determination of homoeriodictyol products 14
Figure S10. Optical purity determination of 7,3'-dihydroxy-4'-methoxyflavanone 15
Table S1. Active pocket alignment of potential bacterial CHIs 16
Table S2. Specific activity of potential bacterial CHIs and CHI_{era} mutants 18
Table S3. Primer list for mutagenesis of CHI_{era} 19
Figure S11. ^{1}H NMR spectrum (600 MHz) of Naringenin (1b) 20
Figure S12. ^{13}C NMR spectrum (151 MHz) of Naringenin (1b) 20
Figure S13. ^{1}H NMR spectrum (600 MHz) of Eriodictyol (2b) 21
Figure S14. ^{13}C NMR spectrum (151 MHz) of Eriodictyol (2b) 21
Figure S15. ^{1}H NMR spectrum (600 MHz) of Homoeriodictyol (3b) 22
Figure S16. ^{13}C NMR spectrum (151 MHz) of Homoeriodictyol (3b) 22
Figure S17. ^{1}H NMR spectrum (600 MHz) of Hesperetin (4b) 23
Figure S18. ^{13}C NMR spectrum (151 MHz) of Hesperetin (4b) 23
Figure S19. ^{1}H NMR spectrum (600 MHz) of 4-*O*-methylbutein (5a) and 7,3'-dihydroxy-4'-methoxyflavanone (5b) 24
Figure S20. ^{13}C NMR spectrum (151 MHz) of 4-*O*-methylbutein (5a) and 7,3'-dihydroxy-4'-methoxyflavanone (5b) 24
Figure S21. DEPT-135 NMR spectrum (151 MHz) of 4-*O*-methylbutein (5a) and 7,3'-dihydroxy-4'-methoxyflavanone (5b) 25
Figure S22. ^{1}H,^{1}H-COSY NMR spectrum (600 MHz) of 4-*O*-methylbutein (5a) and 7,3'-dihydroxy-4'-methoxyflavanone (5b) 25
Figure S23. ^{1}H,^{13}C HSQC NMR spectrum (600 MHz / 151 MHz) of 4-*O*-methylbutein (5a) and 7,3'-dihydroxy-4'-methoxyflavanone (5b) 26
Figure S24. ^{1}H,^{13}C HMBC NMR spectrum (600 MHz / 151 MHz) of 4-*O*-methylbutein (5a) and 7,3'-dihydroxy-4'-methoxyflavanone (5b) 26
Table S4. Correlations identified via 2D-NMR experiments of 7,3'-dihydroxy-4'-methoxyflavanone 27
Table S5. Assignment details for 7,3'-dihydroxy-4'-methoxyflavanone 27
Table S6. Correlations identified via 2D-NMR experiments of 4-*O*-methylbutein 28
Table S7. Assignment details for 4-*O*-methylbutein 28
Table S8. Results of qNMR experiments on flavanone and chalcon samples 29
References 30
Author contributions 30

Experimental Procedures

Materials

Naringenin chalcone, eriodictyol chalcone, homoeriodictyol chalcone and 4-*O*-methylbutein were provided by Symrise AG (Germany). All other chemicals were purchased from Sigma-Aldrich (Germany) unless stated otherwise. Gene synthesis service was offered by BioCat (Germany). The Pfu Plus polymerase kit was purchased from Roboklon (Germany). Restriction enzymes were purchased from New England Biolab (UK). Sequencing service was offered by Eurofins (Germany).

Protein sequence analysis and phylogenetic tree building

The search of homologous protein sequences was carried out with the BLAST algorithm. The protein sequence of CHI_{era} (Genbank access no. AGS82960.1) and the fragment sequences of the catalytic domain (1-143 a.a.) and solvent domain (144-266 a.a.)[1] were used as search queries. The non-redundant protein sequences of Genbank (until April 2019) was used as database for searching. The parameter "maximum target sequences" was adjusted to 20000 to get as many sequences as possible. The data set achieved from the BLAST search was clustered with the CD-HIT tool[2] (http://cd-hit.org) to remove duplicates. The maximum percentage identity was defined as 99%. The sequences that were too long (> 350 amino acids) or too short (< 250 amino acids) in comparison to CHI_{era} were excluded from the sequence list. This final data set contained 88 protein sequences. The default T-coffee algorithm[3] was used to create multisequence alignments. The transitive consistency score represents the reliability of positions in the multisequence alignments and is represented as a number ranged between 0 and 1000, where 1000 means absolute reliability (usually only for identical sequences). The multiple sequence alignment (T-Coffee) shows relatively high homology between the selected sequences (Figure S1). The command line tool IQ-TREE[4] (http://www.iqtree.org/) was used to compute a phylogenetic tree from a multisequence alignment. The default parameters were used for the tree construction. The obtained phylogenetic tree was rooted to midpoint with the FigTree software (http://tree.bio.ed.ac.uk/software/figtree/) to ensure a better comparability (Figure 1, a full version is included in Figure S2).

Protein sequence–structure analysis

The protein sequences in the phylogenetic tree were submitted to SWISS-MODEL for protein structure simulation. The obtained protein structures were aligned by PyMOL software (Schrödinger, USA) to identify the overall structure similarity and active pockets.

Mutagenesis of CHI_{era}

The site-directed mutagenesis was carried out with the QuikChange® method. The primers were designed with the webtool QuikChange® Primer Design offered by Agilent (USA) and are listed in Table S3. The PCR protocol carried out using the Pfu Plus polymerase from Roboklon (Germany) is as follows: 98°C 30 sec, (98°C 10 sec, 68°C 10 sec, 72°C 3.5 min)×15 cycle, 72°C 10 min. The PCR products were digested by DpnI and then transformed into *E. coli* (BL21DE3) chemical competent cells directly by heat shock. The mutations were confirmed by sequencing.

Expression and purification of bacterial CHIs

The synthetic genes and CHI mutant genes were inserted into the vector pET28b with NdeI and BamHI restriction enzymes at the 5'- and 3'-terminii, respectively. The obtained plasmids were transformed into *E. coli* (BL21DE3) chemical competent cells by heat shock. The recombinant *E. coli* cells were cultured in a 1 L LB medium at 37℃ until OD reached 0.5 and induced by adding IPTG (0.1 mM) for protein expression at 28℃. The cells were harvested by centrifugation at 4000×*g* for 15 min. The harvested cells were lysed with 0.5 mg/mL lysozyme (Sigma-Aldrich), 0.4 U/mL benzonase (Sigma-Aldrich) and BugBuster (Merck) at room temperature for 0.5 h to obtain a crude cell extract. The crude extract was centrifugated at 10000×*g* for 30 min to remove the pallet. The supernatant was used for further purification. Recombinant proteins were purified by affinity chromatography with a HisTrap HP 5 mL column (GE Healthcare). The target proteins were eluted by 150 mM imidazole with 20 mM PBS (pH 7.4) and 500 mM NaCl. Buffer exchange with 50 mM PBS (pH 7.5) was achieved by ultrafiltration with Amicon Ultra-15 (Merck). The concentration of proteins was measured by NanoDrop (ThermoFisher).

Assay for bacterial CHIs[5]

The standard curve of the assay was determined by using a series of chalcone concentrations (0-200 µM) in 50 mM PBS (pH 8) in a 96-well plate. The relationship between concentration of chalcones and absorbance was measured at 384 nm[6] by using a plate reader (Tecan, Switzerland) to obtain the standard curve as y=ax+b. The reactions were performed in 96-well plates at 25°C (200 µL per well). The reaction mixture contained 50 mM PBS (pH 8), 100 µM chalcone and CHIs at varying enzyme concentration according to their activity towards different substrates. The absorbance decrease was measured at 384 nm by using a plate reader. The specific activity was calculated according to the following formula:

$$\text{specific activity } [\text{U mg}^{-1}] = \frac{\text{slope} \times 10^{3} [\mu\text{M s}^{-1}] \times 60 \ [\text{s}] \times 200 \times \ 10^{-6} \ [\text{L}]}{\text{amount of CHI [mg]}}$$

1 U is defined as the amount of enzyme required to convert 1 µmol of chalcone to flavanone under the assay conditions.

Stereoselectivity determination of CHI_{era}

The reactions for stereoselectivity determination of CHI_{era} were carried out in triplicates by adding 100 µM of substrate (naringenin chalcone, hesperetin chalcone, eriodictyol chalcone, homoeriodictyol chalcone or 4-*O*-methylbutein) into 50 mM PBS (pH 8.0) containing 1 mg/mL of enzyme (CHI_{era}, CHI_{mut5} and CHI_{mut11} for naringenin chalcone, eriodictyol chalcone, homoeriodictyol chalcone

and hesperetin chalcone; CHI_{mut11} for 4-*O*-methylbutein). A control reaction was carried out by incubating the respective substrate in buffer without enzyme. Total volume of the reactions was 100 µL. The reactions with naringenin chalcone, hesperetin chalcone, eriodictyol chalcone and homoeriodictyol chalcone were stopped with 100 µl methanol after decolorization (~1 min). The reaction with 4-*O*-methylbutein did not decolorize and was stopped after 4 h of incubation. After that, samples were separated and analyzed on a Waters UPC²-QDA system using a Waters Trefoil Amylose 1 column (2.5 µm, 2.1 mm x 150 mm). Naringenin/Naringenin chalcone reactions were separated isocratically with 75 % CO_2 / 25 % methanol, 2000 psi, flow rate 2.0 ml/min, 40°C. Hesperetin chalcone reactions were separated isocratically with 70 % CO_2 / 30 % methanol, 2000 psi, flow rate 1.6 ml/min, 40°C. Eriodictyol chalcone reactions were separated isocratically with 80 % CO_2 / 20 % methanol, 2500 psi, flow rate 2.0 ml/min, 40°C. Homoeriodictyol chalcone reactions were separated isocratically with 85 % CO_2 / 15 % methanol, 2000 psi, flow rate 1.6 ml/min, 40°C. 4-*O*-methylbutein reactions were separated with a gradient (99 % CO_2 / 1 % methanol -> 70 % CO_2 / 30 % methanol (0-19 min)), 2000 psi, flow rate 1.6 ml/min, 40°C.

NMR experiments

The quantification of the products was carried out using a Bruker Ascend 600 (B_0 = 14.1 T, Bruker Biospin, Rheinstetten, Germany) with resonance frequencies of 600 MHz for ^{1}H and 151 MHz for ^{13}C, respectively. The solvent DMSO-d6 (δ(^{1}H) = 2.50 ppm, δ(^{13}C) = 39.52 ppm) was used for chemical shifts referencing for all spectra. The samples contained traces of *n*-hexane, ethyl acetate and acetone which are denoted in the corresponding spectra. *qNMR*: Three samples were prepared for each sample material using an amount of ~2-7 mg sample and quantification reference, respectively. To also minimize errors due to the integration procedure and the bias of the spectrometer all samples were measured three times and analyzed. 1,2,4,5-Tetrachloro-3-nitrobenzene (TraceCERT®) with a purity of 99,82% was used as quantification reference (QR) in all analysis. For each of the flavanones (**1b**-**5b**) the area of peak ($I_{Analyte}$) of the CH group at position 2 at ~5.4 ppm was used and correlated with the area peak of the QR (I_{QR}) under considering the molar masses of the analyte ($M_{Analyte}$) and the QR (M_{QR}) as well as the purity of the QR (P_{QR}). The formula is denoted below and was provided with the TraceCERT® certificate of the QR. For the chalcone **5a** (4-*O*-methylbutein) the signals of 2 protons around 7.7 ppm were used as the analyte integral ($I_{Analyte}$). According to the sample structure the number of nuclei per molecule represented by the integrated signals was $N_{Analyte} = N_{QR} = 1$ for the flavanones (5.4 ppm) and $N_{QR} = 2$ for the chalcone 4-*O*-methylbutein (**5a**).

$$P_{Sample} = \frac{I_{Analyte} N_{QR} M_{Analyte} m_{QR} P_{QR}}{I_{QR} N_{Analyte} M_{QR} m_{Sample}}$$

Enzymatic synthesis of naringenin (1b) by CHI_{era}

The enzymatic reactions were carried out by dropping 35 mg of naringenin chalcone (in 1 mL MeOH stock) into 100 mL PBS (50 mM, pH 8.0) containing 2 mg of CHI_{era}. The reaction was stopped when there was no color change in the reaction solution (~10 min). The product was extracted by 200 mL ethyl acetate directly after the reaction and dried by magnesium sulfate anhydrous. The solvent and water were removed by using a rotary evaporator. The product (41.9 mg yellow powder) was obtained after lyophilization for 2 days. The chemical structure and purity (88.2 ± 7.1%) of the product was determined by NMR as descripted above. The yield was ~100%. ^{1}H NMR (600 MHz, 298.0 K, DMSO) δ = 12.15 (s, 1H), 10.78 (s, 1H), 9.58 (s, 1H), 7.31 (m, 2H), 6.79 (m, 2H), 5.88 (s, 2H), 5.44 (dd, *J*=12.8, 3.0, 1H), 3.26 (dd, *J*=17.1, 12.8, 1H), 2.68 (dd, *J*=17.1, 3.0, 1H). ^{13}C NMR (151 MHz, 298.0 K, DMSO) δ = 196.4, 166.7, 163.5, 163.0, 157.7, 128.9, 128.4, 115.2, 101.8, 95.8, 95.0, 78.4, 42.0.

Enzymatic synthesis of eriodictyol (2b) by CHI_{era}Mut5

The enzymatic reactions were carried out by dropping 30 mg of eriodictyol chalcone (in 1 mL MeOH stock) into 100 mL PBS (50 mM, pH 8.0) containing 2.7 mg of CHI_{era}Mut5. The reaction was stopped by adding 200 mL MeOH when there was no color change in the reaction solution (~10 min). The reaction solution was filtered to remove the precipitate. The solvent and water were removed by using a rotary evaporator. The product (95.5 mg yellow powder) was obtained after lyophilization for 2 days. The chemical structure and purity (37.8 ± 3.5%) of the product was determined by NMR as descripted above. The yield was ~100%. ^{1}H NMR (600 MHz, 298.0 K, DMSO) δ = 12.12 (s, 1H), 11.07 (s, 1H), 9.16 (s, 2H), 6.89 (d, *J*=2.1, 1H), 6.77 (d, *J*=8.1, 1H), 6.72 (dd, *J*=8.1, 2.1, 1H), 5.95 (d, *J*=2.1, 1H), 5.94 (d, *J*=2.1, 1H), 5.36 (dd, *J*=12.5, 3.1, 1H), 3.16 (dd, *J*=17.1, 12.6, 1H), 2.66 (dd, *J*=17.1, 3.1, 1H). ^{13}C NMR (151 MHz, 298.0 K, DMSO) δ = 196.3, 166.9, 163.4, 162.8, 145.7, 145.2, 129.4, 117.9, 115.5, 114.5, 101.7, 95.9, 95.1, 78.4, 42.1.

Enzymatic synthesis of homoeriodictyol (3b) by CHI_{era}Mut5

The enzymatic reactions were carried out by dropping 30 mg of homoeriodictyol chalcone (in 1 mL DMSO stock) into 100 mL PBS (50 mM, pH 8.0) containing 3 mg of CHI_{era}Mut5. The reaction was stopped when there was no color change in the reaction solution (~10 min). The product was extracted by 200 mL ethyl acetate directly after the reaction and dried by magnesium sulfate anhydrous. The solvent and water were removed by using a rotary evaporator. The product (48.5 mg yellow powder) was obtained after lyophilization for 2 days. The chemical structure and purity (63.4 ± 5.2%) of the product was determined by NMR as descripted above. The yield was ~100%. ^{1}H NMR (600 MHz, 298.0 K, DMSO) δ = 12.15 (s, 1H), 10.78 (s, 1H), 9.13 (s, 1H), 7.09 (d, *J*=2.0, 1H), 6.90 (dd, *J*=8.1, 2.1, 1H), 6.79 (d, *J*=8.1, 1H), 5.89 (d, *J*=2.1, 1H), 5.88 (d, *J*=2.1, 1H), 5.43 (dd, *J*=12.9, 3.0, 1H), 3.78 (s, 3H), 3.32 (dd, *J*=17.1, 12.9, 1H), 2.68 (dd, *J*=17.1, 3.0, 1H). ^{13}C NMR (151 MHz, 298.0 K, DMSO) δ = 196.4, 166.6, 163.5, 162.9, 147.5, 147.0, 129.4, 119.7, 115.2, 111.2, 101.7, 95.8, 95.0, 78.7, 55.7, 42.1.

Enzymatic synthesis of hesperetin (4b) by CHI_{era}Mut11

The enzymatic reactions were carried out by dropping 35 mg of hesperetin chalcone (in 1 mL MeOH stock) into 100 mL PBS (50 mM, pH 8.0) containing 4.5 mg of CHI_{era}Mut11. The reaction was stopped when there was no color change in the reaction solution (~10

min). The product was extracted by 200 mL ethyl acetate directly after the reaction and dried by magnesium sulfate anhydrous. The solvent and water were removed by using a rotary evaporator. The product (46.3 mg yellow powder) was obtained after lyophilization for 2 days. The chemical structure and purity (81.3 ± 8.2%) of the product was determined by NMR as descripted above. The yield was ~100%. ^{1}H NMR (600 MHz, 298.0 K, DMSO) δ = 12.13 (s, 1H), 10.79 (s, 1H), 9.09 (s, 1H), 6.93 (d, *J*=8.3, 1H), 6.92 (d, *J*=2.2, 1H), 6.87 (dd, *J*=8.3, 2.2, 1H), 5.89 (d, *J*=2.1, 1H), 5.88 (d, *J*=2.1, 1H), 5.43 (dd, *J*=12.4, 3.2, 1H), 3.77 (s, 3H), 3.20 (dd, *J*=17.1, 12.4, 1H), 2.71 (dd, *J*=17.1, 3.2, 1H). ^{13}C NMR (151 MHz, 298.0 K, DMSO) δ = 196.2, 166.6, 163.5, 162.8, 147.9, 146.5, 131.1, 117.7, 114.1, 112.0, 101.8, 95.8, 95.0, 78.2, 55.7, 42.1.

Enzymatic synthesis of 7,3'-dihydroxy-4'-methoxyflavanone (5b) by CHI$_{era}$Mut11

The enzymatic reactions were carried out by dropping 30 mg of 4-*O*-methylbutein (in 1 mL DMSO stock) into 100 mL PBS (50 mM, pH 8.0) containing 16.8 mg of CHI$_{era}$Mut11. The reaction was stopped after 2 days. The product was extracted by 200 mL ethyl acetate directly after the reaction and dried by magnesium sulfate anhydrous. The solvent and water were removed by using a rotary evaporator. The product (52.3 mg yellow powder) was obtained after lyophilization for 2 days. The chemical structure and purity (48.9 ± 3.8%) of the product was determined by NMR as descripted above. The yield was 85%.

7,3'-dihydroxy-4'-methoxyflavanone (5b) ^{1}H NMR (600 MHz, DMSO) δ = 10.56 (s, 1H) 9.07 (s, 1H), 7.64 (d, *J*=8.7, 1H), 6.93 (d, *J*=2.1), 6.93 (d, *J*=8.3, 1H), 6.88 (dd, *J*=8.3, 2.1, 1H), 6.50 (dd, *J*=8.7, 2.2, 1H), 6.34 (d, *J*=2.2, 1H), 5.43 (dd, *J*=12.5, 3.0, 1H), 3.77 (s, 3H), 3.05 (dd, *J*=16.7, 12.5, 1H), 2.65 (dd, *J*=16.7, 3.0, 1H). ^{13}C NMR (151 MHz, 298.0 K, DMSO) δ = 189.9, 164.6, 163.0, 147.8, 146.4, 131.6, 128.4, 117.6, 114.0, 113.6, 112.0, 110.5, 102.6, 78.8, 55.67, 43.3.

4-*O*-methylbutein (5a) ^{1}H NMR (600 MHz, DMSO) δ = 13.53 (s, 1H), 10.67 (s, 1H), 9.14 (s, 1H), 8.17 (d, *J*=8.9, 1H), 7.73 (d, *J*=15.2, 1H), 7.68 (d, *J*=15.3, 1H), 7.34 (d, *J*=2.1, 1H), 7.31 (dd, *J*=8.4, 2.1, 1H), 7.00 (d, *J*=8.4, 1H), 6.41 (dd, *J*=8.9, 2.4, 1H), 6.28 (d, *J*=2.4, 1H), 3.84 (s, 3H). ^{13}C NMR (151 MHz, 298.0 K, DMSO) δ = 191.5, 165.8, 165.0, 150.4, 146.7, 144.3, 133.0, 127.6, 122.3, 118.4, 115.1, 113.0, 111.9, 108.1, 102.6, 55.71.

Additional Results and Discussion

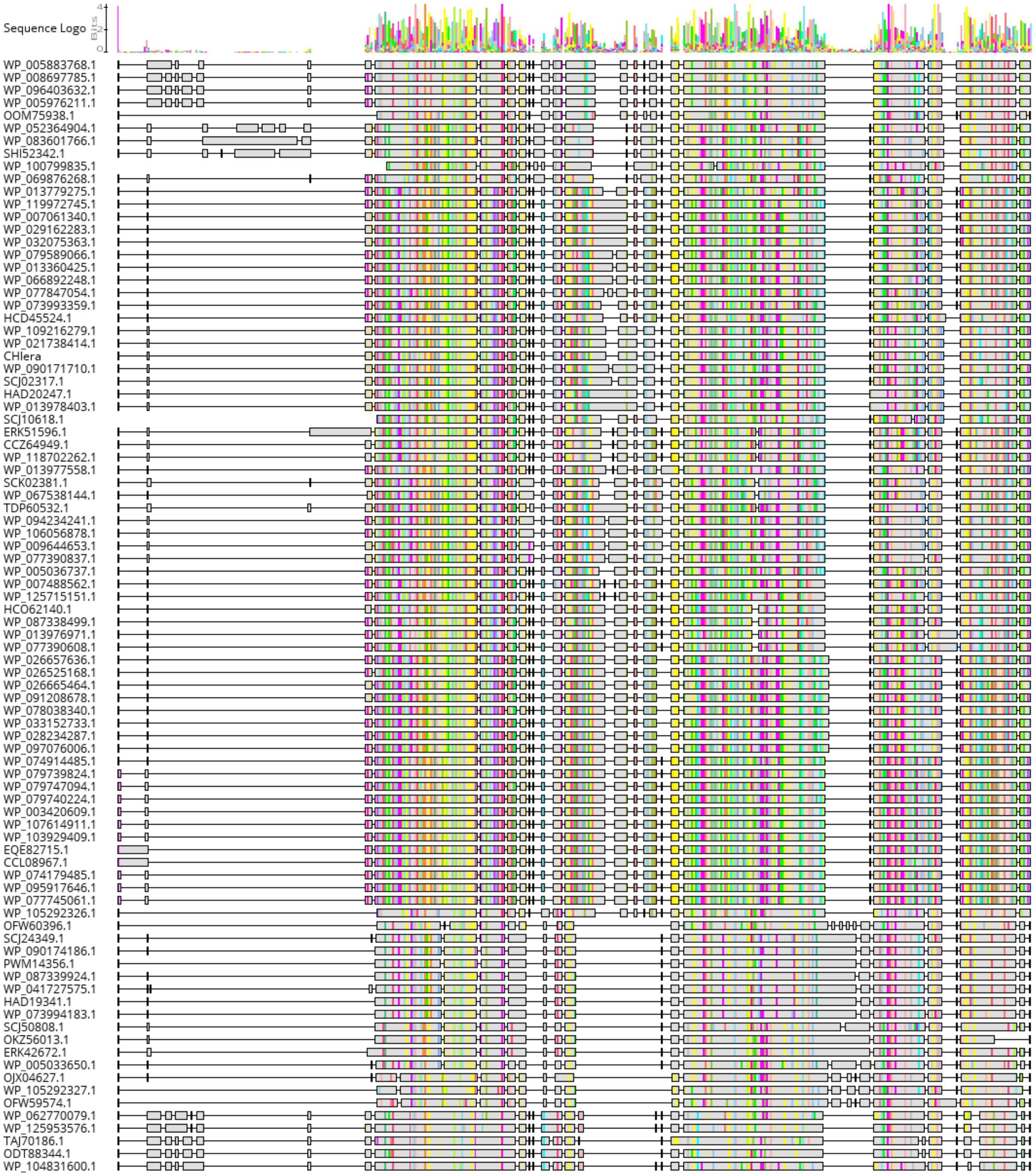

Figure S1. Full protein sequence alignment of potential bacterial CHIs.

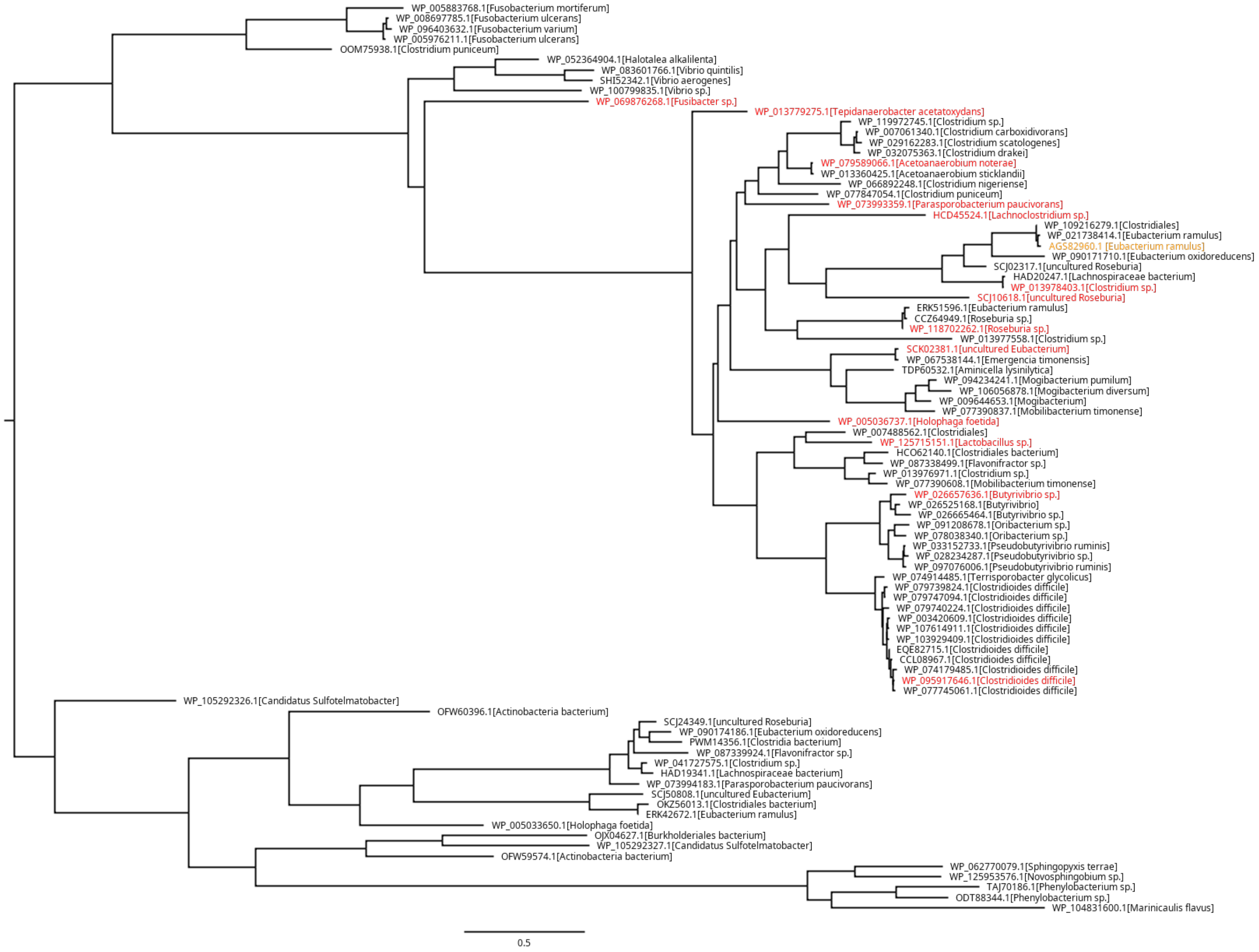

Figure S2. Full phylogenetic tree of potential bacterial CHIs. The proteins selected for activity determination are highlighted in red. CHI$_{era}$ is highlighted in yellow.

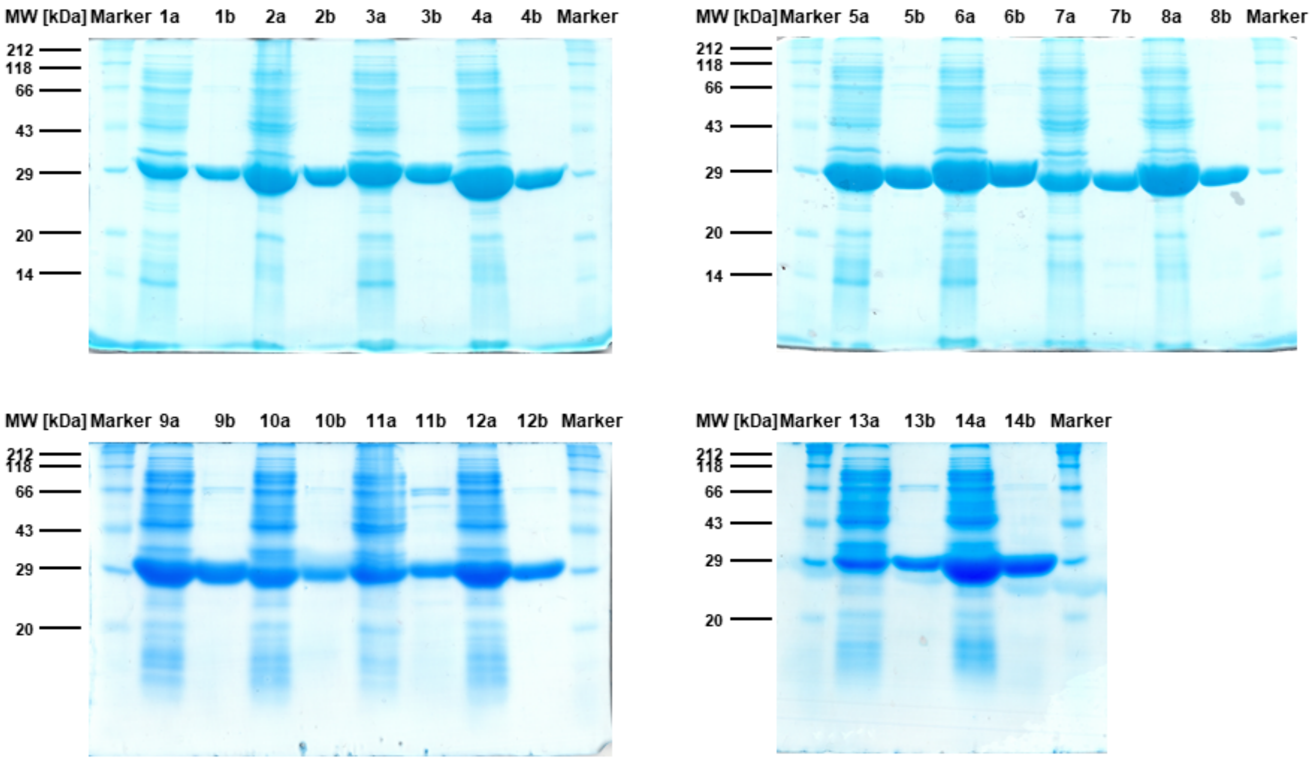

Figure S3. SDS-PAGE for expression and purification of potential bacterial CHIs. The protein samples of CHI1-CHI13 are labelled as 1-13 in order. CHI_{era} is labelled as 14. The supernatant of cell lysate and purified recombinant enzymes are labelled as a and b, respectively.

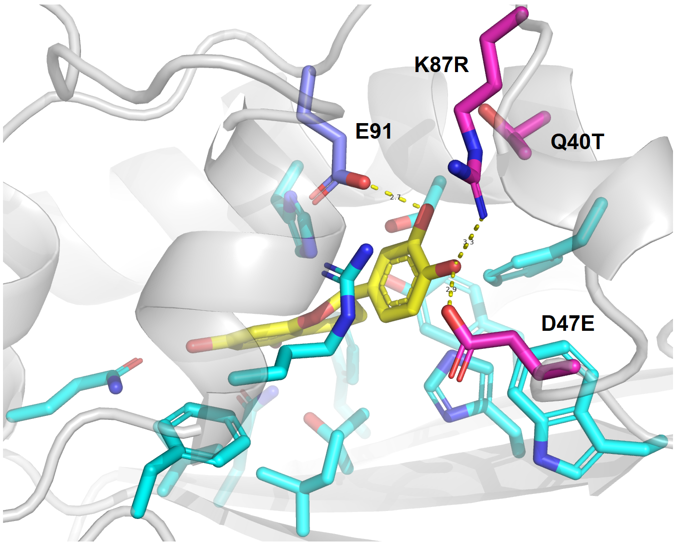

Figure S4. Simulation of the active site pocket of CHI$_{era}$Mut5. The simulated protein structure was created by SWISS-MODEL with use of the X-ray crystal structure of CHI$_{era}$ (PDB ID: 4D06) as template. The mutated residues are labelled in red. Yellow: (*S*)-eriodictyol. The graphic was created with PyMOL software.

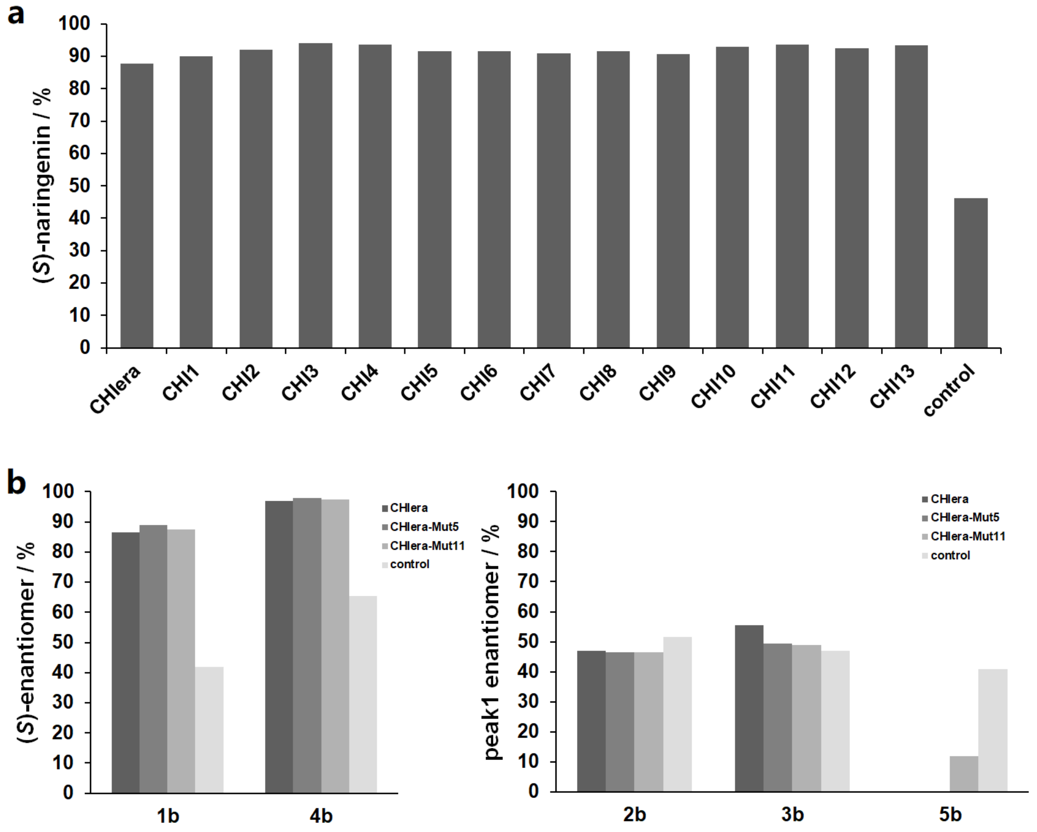

Figure S5. Stereoselectivity determination of bacterial CHIs. (**a**) Optical purity determination of the naringenin products catalyzed by bacterial CHIs with naringenin chalcone as substrate. All bacterial CHIs showed (*S*)-stereoselectivity. The control reaction was carried out by incubating chalcone in buffer without enzyme. In comparison to the controls, all wild-type CHIs showed (*S*)-stereoselectivity towards naringenin chalcone. (**b**) Optical purity determination of the naringenin, hesperetin, eriodictyol, and homoeriodictyol products catalyzed by CHI_{era}, CHI_{era}-Mut5, and CHI_{era}-Mut11, and 7,3'-dihydroxy-4'-methoxyflavanone product catalyzed by CHI_{era}-Mut11. The control reaction was carried out by incubating chalcone in buffer without enzyme. In comparison to the controls, CHI_{era}, CHI_{era}-Mut5, and CHI_{era}-Mut11 showed (*S*)-stereoselectivity towards naringenin chalcone and hesperetin chalcone, but almost no stereoselectivity to eriodictyol chalcone and homoeriodictyol chalcone. CHI_{era}-Mut11 showed high stereoselectivity towards 4-*O*-methylbutein. However, due to lack of standard compounds, (*R*)- and (*S*)-enantiomers cannot be assigned to peak 1 or 2. **1b**: naringenin, **2b**: eriodictyol, **3b**: homoeriodictyol, **4b**: hesperetin, **5b**: 7,3'-dihydroxy-4'-methoxyflavanone.

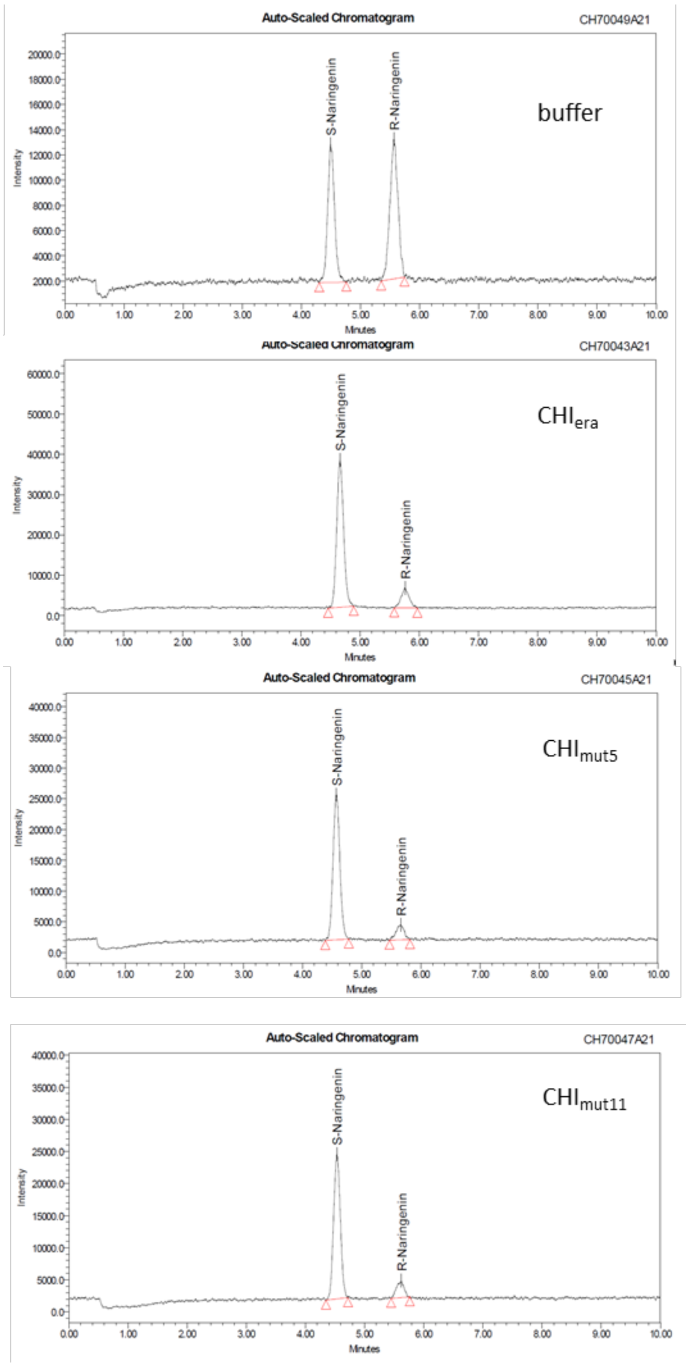

Figure S6. Optical purity determination of naringenin products obtained by bacterial CHIs and CHI mutants with naringenin chalcone as substrate. (*S*)-Naringenin and (*R*)-naringenin elution times have been determined using standard compounds.

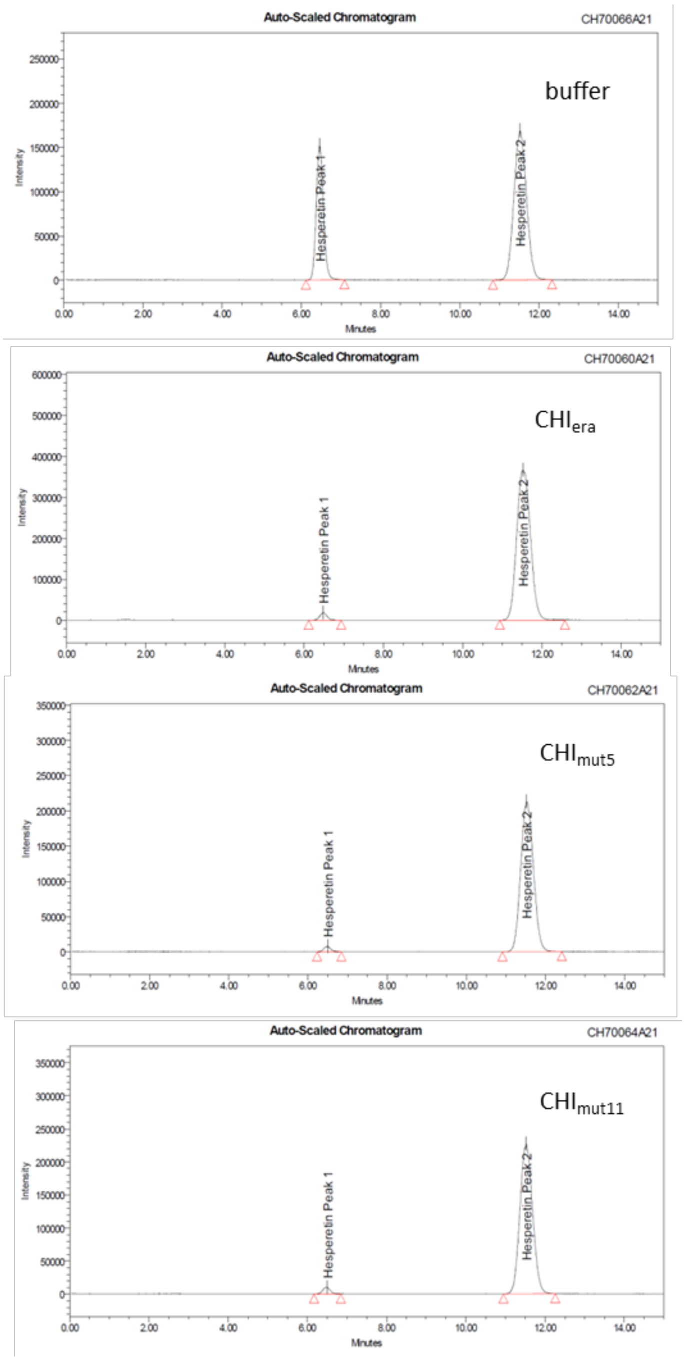

Figure S7. Optical purity determination of hesperetin products obtained by bacterial CHIs and CHI mutants with hesperetin chalcone as substrate. Hesperetin peak 1 confers to (*R*)-hesperetin and hesperetin peak 2 confers to (*S*)-hesperetin (determined via standard compounds).

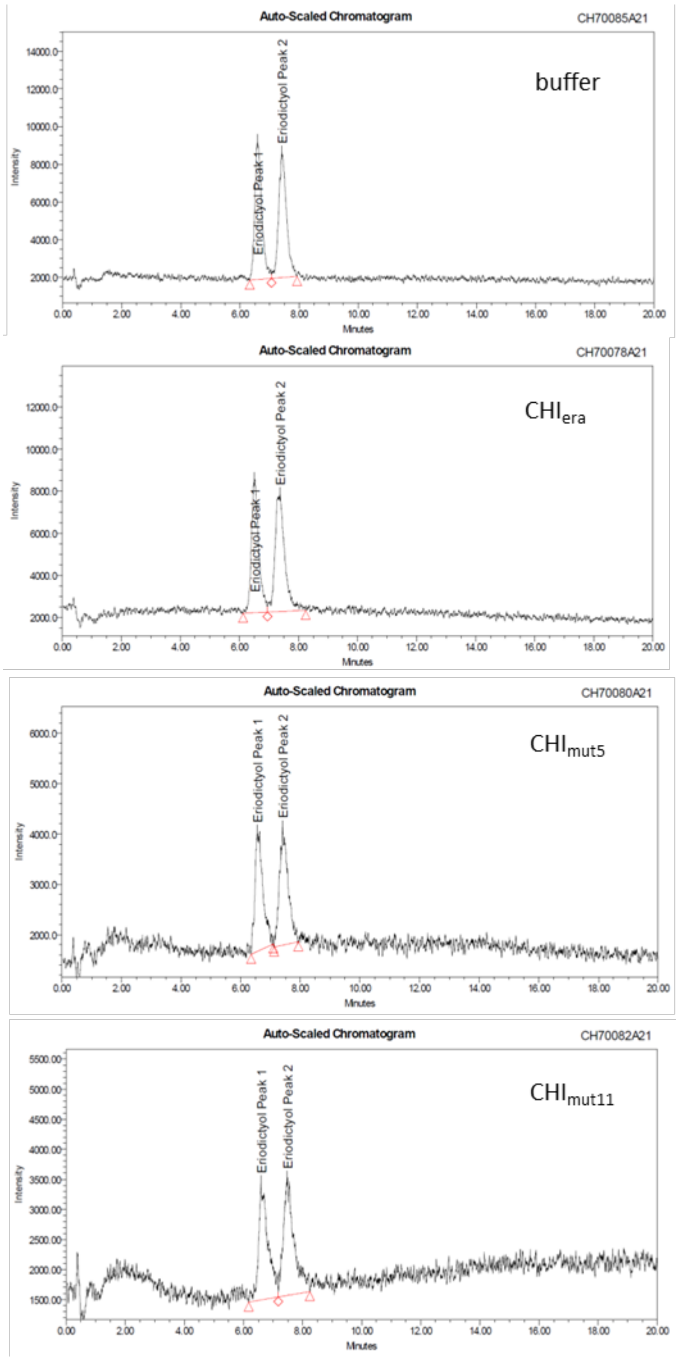

Figure S8. Optical purity determination of eriodictyol products obtained by bacterial CHIs and CHI mutants with eriodictyol chalcone as substrate. Due to lack of standard compounds, (*R*)- and (*S*)-enantiomers cannot be assigned to peak 1 or 2.

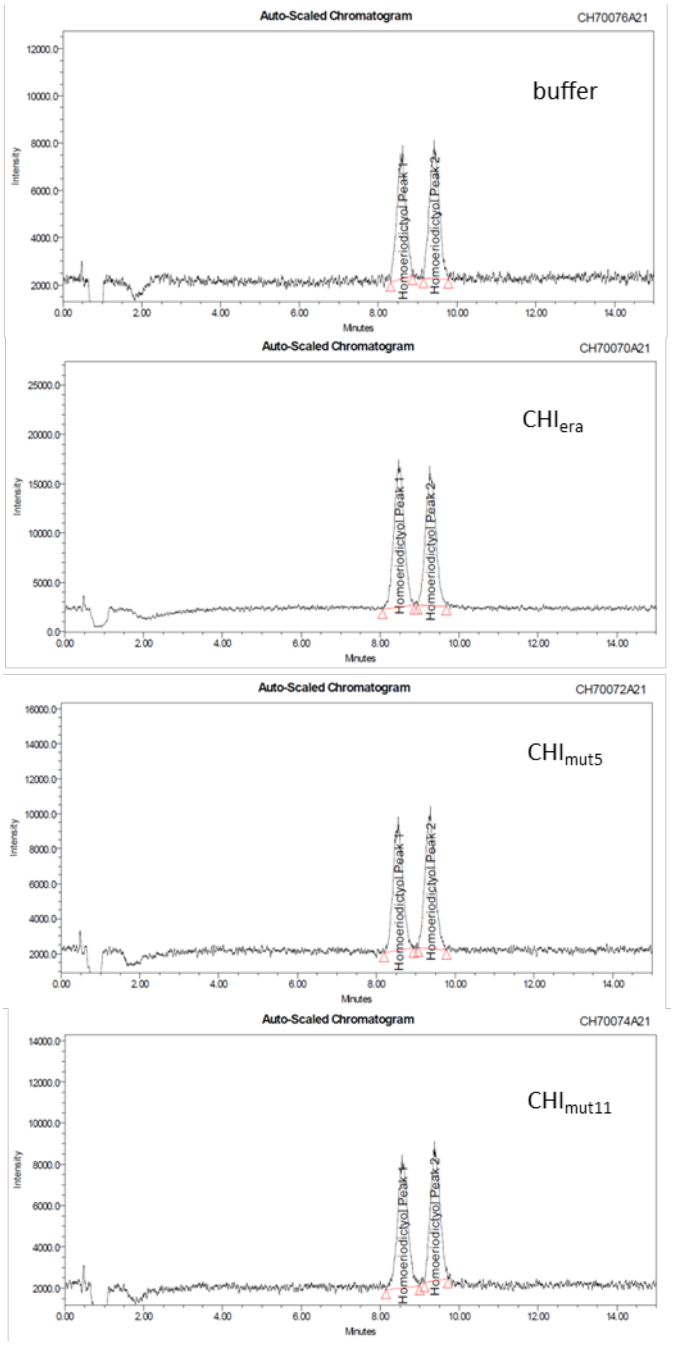

Figure S9. Optical purity determination of homoeriodictyol products obtained by bacterial CHIs and CHI mutants with homoeriodictyol chalcone as substrate. Due to lack of standard compounds (*R*)- and (*S*)-enantiomers cannot be assigned to peak 1 or 2.

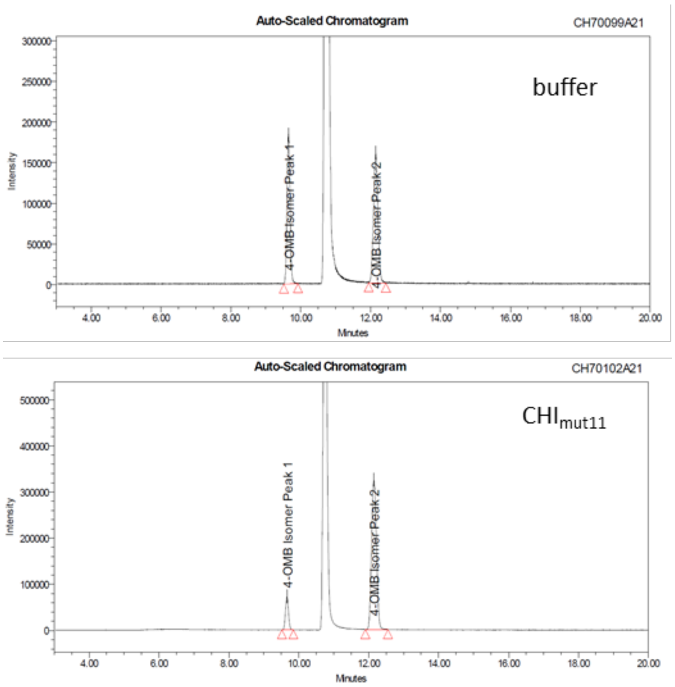

Figure S10. Optical purity determination of 7,3'-dihydroxy-4'-methoxyflavanone product obtained by CHI_{era}-Mut11 with 4-*O*-methylbutein as substrate. The peak at retention time of 10.5 min refers to 4-*O*-methyl butein, peaks at 9.7 min and 12.1 min refer to the (*R*)- and (*S*)-enantiomers of 7,3'-dihydroxy-4'-methoxyflavanone. Due to lack of standard compounds, (*R*)- and (*S*)-enantiomers cannot be assigned to peak 1 or 2.

Table S1. Active pocket alignment of potential bacterial CHIs.

Branch	Access no.	A-ring								C-ring				B-ring								
		101	69	71	135	29	14	50	12	125	48	33	122	79	87	40	125	41	73	37	91	75
1	WP_005883768.1	I	R	T	A	L	L	T	L	-	Y	**H**	D	P	T	I	-	N	H	T	E	W
	WP_008697785.1	I	R	T	A	L	L	T	L	-	Y	**H**	D	P	T	I	-	N	H	T	E	W
	WP_096403632.1	I	R	T	A	L	L	T	L	-	Y	**H**	D	P	T	I	-	N	H	T	E	W
	WP_005976211.1	I	R	T	A	L	L	T	L	-	Y	**H**	D	P	T	I	-	N	H	T	E	W
	OOM75938.1	I	R	T	P	L	L	T	L	E	Y	**H**	D	P	S	Q	E	N	H	T	E	W
2	WP_052364904.1	T	I	T	T	L	V	T	I	Q	Y	**H**	D	P	L	Q	Q	F	H	S	E	W
	WP_083601766.1	T	I	T	T	L	V	T	I	H	Y	**H**	D	P	L	Q	H	F	H	S	E	W
	SHI52342.1	T	I	T	T	L	V	T	I	H	Y	**H**	D	P	L	Q	H	F	H	S	E	W
	WP_100799835.1	T	I	T	T	L	V	T	-	E	Y	**H**	L	P	V	Q	E	F	H	S	E	W
3	WP_069876268.1	T	I	T	I	L	N	T	I	G	Y	**H**	T	I	L	Q	G	F	H	S	E	W
4	WP_013779275.1	Q	Q	T	F	L	V	F	I	R	Y	**H**	D	P	K	Q	R	F	H	S	E	W
5	WP_119972745.1	Q	Q	T	F	L	V	F	I	R	Y	**H**	D	P	K	Q	R	F	H	S	E	W
	WP_007061340.1	Q	Q	T	F	L	V	F	I	R	Y	**H**	D	P	K	Q	R	F	H	S	E	W
	WP_029162283.1	Q	Q	T	F	L	V	F	I	R	Y	**H**	D	P	K	Q	R	F	H	S	E	W
	WP_032075363.1	Q	Q	T	F	L	V	F	I	R	Y	**H**	D	P	K	Q	R	F	H	S	E	W
	WP_079589066.1	Q	Q	T	F	L	V	F	I	R	Y	**H**	D	P	K	Q	R	F	H	S	E	W
	WP_013360425.1	Q	Q	T	F	L	V	F	I	R	Y	**H**	D	P	K	Q	R	F	H	S	E	W
	WP_066892248.1	Q	Q	T	F	L	V	F	I	R	Y	**H**	N	M	K	Q	R	F	H	S	E	W
	WP_077847054.1	Q	Q	T	F	L	V	F	I	R	Y	**H**	D	P	K	Q	R	F	H	S	E	W
	WP_073993359.1	Q	Q	T	F	L	V	F	I	R	Y	**H**	D	P	K	Q	R	F	H	S	E	W
6	HCD45524.1	Q	Q	T	F	L	V	F	I	R	Y	**H**	D	I	K	Q	R	F	H	S	E	W
7	WP_109216279.1	Q	Q	T	F	L	V	F	I	R	Y	**H**	N	D	K	Q	R	F	H	S	E	W
	WP_021738414.1	Q	Q	T	F	L	V	F	I	R	Y	**H**	N	D	K	Q	R	F	H	S	E	W
	CHI_{era}	Q	Q	T	F	I	V	F	I	R	Y	**H**	N	D	K	Q	R	F	H	S	E	W
	WP_090171710.1	Q	Q	T	F	L	V	F	I	R	Y	**H**	N	D	K	Q	R	F	H	S	E	W
	SCJ02317.1	Q	Q	T	F	L	V	F	I	R	Y	**H**	N	D	K	Q	R	F	H	S	E	W
	HAD20247.1	Q	Q	T	F	L	V	F	I	R	Y	**H**	N	D	K	Q	R	F	H	SS	E	W
	WP_013978403.1	Q	Q	T	F	L	V	F	I	R	Y	**H**	N	D	K	Q	R	F	H	S	E	W
	SCJ10618.1	Q	Q	T	F	L	V	F	I	R	Y	**H**	D	P	N	K	R	F	H	S	E	W
8	ERK51596.1	Q	Q	T	F	L	V	F	I	R	Y	**H**	E	P	N	K	R	F	H	SS	E	W
	CCZ64949.1	Q	Q	T	F	L	V	F	I	R	Y	**H**	E	P	N	K	R	F	H	S	E	W
	WP_118702262.1	Q	Q	T	F	L	V	F	I	R	Y	**H**	E	P	N	K	R	F	H	S	E	W
	WP_013977558.1	Q	Q	T	F	L	I	F	I	R	Y	**H**	D	P	N	K	R	F	H	SS	E	W
9	SCK02381.1	Q	Q	T	F	L	V	F	I	R	Y	**H**	D	P	K	Q	R	F	H	S	E	W
	WP_067538144.1	Q	Q	T	F	L	V	F	I	R	Y	**H**	D	P	K	Q	R	F	H	S	E	W
	TDP60532.1	Q	Q	T	F	L	V	F	I	R	Y	**H**	D	P	K	Q	R	F	H	S	E	W
	WP_094234241.1	Q	Q	T	F	L	V	F	I	R	Y	**H**	D	P	K	Q	R	F	H	S	E	W
	WP_106056878.1	Q	Q	T	F	L	V	F	I	R	Y	**H**	D	P	K	Q	R	F	H	S	E	W
	WP_009644653.1	Q	Q	T	F	L	V	F	I	R	Y	**H**	D	P	K	Q	R	F	H	S	E	W
	WP_077390837.1	Q	Q	T	F	L	V	F	I	R	Y	**H**	D	P	K	Q	R	F	H	S	E	W
10	WP_005036737.1	Q	Q	T	F	L	V	F	I	R	Y	**H**	D	P	K	Q	R	F	H	S	E	W
11	WP_007488562.1	Q	Q	T	F	L	V	F	I	R	Y	**H**	D	E	K	N	R	F	H	S	E	W
	WP_125715151.1	Q	Q	T	F	L	V	F	I	R	Y	**H**	D	E	N	K	R	F	H	S	E	W
	HCO62140.1	Q	Q	T	F	L	V	F	I	R	Y	**H**	D	E	N	K	R	F	H	S	E	W
	WP_087338499.1	Q	Q	T	F	L	V	F	I	R	Y	**H**	D	E	N	K	R	F	H	S	E	W
	WP_013976971.1	Q	Q	T	F	L	V	F	I	R	Y	**H**	D	E	N	K	R	F	H	S	E	W
	WP_077390608.1	Q	Q	T	F	L	V	F	I	R	Y	**H**	D	E	N	K	R	F	H	S	E	W
12	WP_026657636.1	Q	Q	T	F	L	V	F	I	R	Y	**H**	D	E	N	K	R	F	H	S	E	W
	WP_026525168.1	Q	Q	T	F	L	V	F	I	R	Y	**H**	D	E	N	K	R	F	H	S	E	W
	WP_026665464.1	Q	Q	T	F	L	V	F	I	R	Y	**H**	D	E	N	K	R	F	H	S	E	W
	WP_091208678.1	Q	Q	T	F	L	V	F	I	R	Y	**H**	D	E	N	K	R	F	H	S	E	W
	WP_078038340.1	Q	Q	T	F	L	V	F	I	R	Y	**H**	D	E	N	K	R	F	H	S	E	W
	WP_033152733.1	Q	Q	T	F	L	V	F	I	R	Y	**H**	D	E	N	K	R	F	H	S	E	W
	WP_028234287.1	Q	Q	T	F	L	V	F	I	R	Y	**H**	D	E	N	K	R	F	H	S	E	W
	WP_097076006.1	Q	Q	T	F	L	V	F	I	R	Y	**H**	D	E	N	K	R	F	H	S	E	W
13	WP_074914485.1	Q	Q	T	F	L	V	F	I	R	Y	**H**	D	E	R	N	R	F	H	S	E	W
	WP_079739824.1	Q	Q	T	F	L	V	F	I	R	Y	**H**	D	E	R	N	R	F	H	S	E	W

	WP_079747094.1	Q	Q	T	F	L	V	F	I	R	Y	**H**	D	E	R	N	R	F	H	S	E	W
	WP_079740224.1	Q	Q	T	F	L	V	F	I	R	Y	**H**	D	E	R	T	R	F	H	S	E	W
	WP_003420609.1	Q	Q	T	F	L	V	F	I	R	Y	**H**	D	E	R	T	R	F	H	S	E	W
	WP_107614911.1	Q	Q	T	F	L	V	F	I	R	Y	**H**	D	E	R	T	R	F	H	S	E	W
	WP_103929409.1	Q	Q	T	F	L	V	F	I	R	Y	**H**	D	E	R	T	R	F	H	S	E	W
	EQE82715.1	Q	Q	T	F	L	V	F	I	R	Y	**H**	D	E	R	T	R	F	H	S	E	W
	CCL08967.1	Q	Q	T	F	L	V	F	I	R	Y	**H**	D	E	R	T	R	F	H	S	E	W
	WP_074179485.1	Q	Q	T	F	L	V	F	I	R	Y	**H**	D	E	R	T	R	F	H	S	E	W
	WP_095917646.1	Q	Q	T	F	L	V	F	I	R	Y	**H**	D	E	R	T	R	F	H	S	E	W
	WP_077745061.1	Q	Q	T	F	L	V	F	I	R	Y	**H**	D	E	R	T	R	F	H	S	E	W
14	WP_105292326.1	L	R	T	P	L	L	S	L	-	Y	**H**	A	P	S	Q	-	N	H	S	E	W
15	OFW60396.1	-	K	H	V	F	H	S	I	-	Y	**H**	-	G	-	N	-	R	N	T	S	S
	SCJ24349.1	-	R	H	A	F	H	L	I	-	Y	**H**	-	L	-	Q	-	Y	N	V	G	A
	WP_090174186.1	-	R	H	A	F	H	L	I	-	Y	**H**	-	E	-	Q	-	Y	N	V	G	A
	PWM14356.1	-	R	H	A	F	H	L	I	-	Y	**H**	-	L	-	H	-	Y	N	V	G	A
	WP_087339924.1	-	R	H	A	F	H	L	I	-	Y	**H**	-	D	-	Q	-	F	N	V	G	A
	WP_041727575.1	-	R	H	A	F	H	M	I	-	Y	**H**	-	Q	-	Q	-	Y	N	V	G	A
16	HAD19341.1	-	R	H	A	F	H	M	I	-	Y	**H**	-	L	-	Q	-	Y	N	V	G	A
	WP_073994183.1	-	R	H	A	F	H	L	I	-	Y	**H**	-	N	-	Q	-	Y	N	V	G	A
	SCJ50808.1	-	R	H	V	F	H	M	I	-	Y	**H**	-	G	-	Q	-	E	N	V	A	G
	OKZ56013.1	-	R	H	V	F	H	M	I	-	Y	**H**	-	G	-	Q	-	E	N	V	G	G
	ERK42672.1	-	R	H	V	F	H	M	I	-	H	**H**	-	G	-	Q	-	E	N	V	G	G
	WP_005033650.1	-	R	H	A	F	H	L	I	-	L	**H**	-	G	-	Q	-	M	N	V	S	V
	OJX04627.1	-	R	T	V	Y	H	S	V	-	H	**H**	-	P	-	R	-	F	G	I	A	W
17	WP_105292327.1	-	R	T	A	Y	H	S	V	-	Y	**H**	-	P	-	R	-	Y	G	I	S	W
	OFW59574.1	-	R	T	I	Y	H	S	V	-	Y	**H**	-	P	-	R	-	Y	T	I	C	W
	WP_062770079.1	-	R	T	S	Y	V	S	I	-	Y	**H**	-	D	D	A	-	Y	I	V	S	D
	WP_125953576.1	-	R	T	S	Y	I	S	I	-	Y	**H**	-	D	G	A	-	Y	I	V	S	D
18	TAJ70186.1	-	R	T	F	Y	I	S	I	-	Y	**H**	-	D	G	A	-	F	I	V	S	D
	ODT88344.1	-	R	T	S	Y	V	S	I	-	Y	**H**	-	D	R	A	-	F	I	V	S	D
	WP_104831600.1	-	R	T	T	Y	I	S	I	-	Y	**H**	-	D	G	A	-	V	I	N	S	D

The proteins selected for activity determination are highlighted in red. “-” means gap in the protein sequence alignment.

Table S2. Specific activity of potential bacterial CHIs and CHI_{era} mutants.

Label	Genbank access no.	Mutation position*							Naringenin chalcone (U/mg)	Eriodictyol chalcone (U/mg)	Homoeriodictyol chalcone (U/mg)	Hesperetin chalcone (U/mg)	4-*O*-Methylbutein (U/mg)
		121	122	79	87	40	125	37					
CHI1	WP_069876268.1	N	T	I	L	Q	G	S	21.3 ± 1.8	18.2 ± 2.0	11.3 ± 1.6	16.7 ± 0.7	0.2 ± 0.0
CHI2	WP_013779275.1	G	D	P	K	Q	R	S	54.8 ± 3.1	25.4 ± 3.1	19.9 ± 5.2	93.5 ± 4.5	0.8 ± 0.3
CHI3	WP_079589066.1	G	D	P	K	Q	R	S	46.6 ± 1.5	34.4 ± 1.7	15.2 ± 7.7	58.5 ± 4.0	0.4 ± 0.0
CHI4	WP_073993359.1	G	D	P	K	Q	R	S	59.9 ± 4.5	82.7 ± 4.0	19.7 ± 1.7	84.5 ± 3.1	0.4 ± 0.0
CHI5	HCD45524.1	G	D	I	K	Q	R	S	16.8 ± 0.5	17.0 ± 1.0	8.1 ± 1.9	3.3 ± 0.2	0.1 ± 0.0
CHI6	WP_013978403.1	G	N	D	K	Q	R	S	68.3 ± 3.3	123.6 ± 12.3	50.2 ± 9.3	6.4 ± 0.4	-
CHI7	SCJ10618.1	G	D	P	N	K	R	S	73.5 ± 4.5	33.9 ± 2.0	22.8 ± 0.7	19.2 ± 0.9	1.2 ± 0.1
CHI8	WP_118702262.1	G	E	P	N	K	R	S	22.7 ± 0.8	69.7 ± 3.5	10.4 ± 1.4	43.0 ± 2.5	0.4 ± 0.0
CHI9	SCK02381.1	G	D	P	K	Q	R	S	23.2 ± 1.0	10.8 ± 1.0	11.4 ± 0.8	5.8 ± 0.2	-
CHI10	WP_005036737.1	G	D	P	K	Q	R	S	32.4 ± 4.0	28.2 ± 1.5	6.2 ± 1.3	36.5 ± 0.8	0.2 ± 0.0
CHI11	WP_125715151.1	G	D	E	N	K	R	S	32.7 ± 2.6	66.2 ± 4.2	21.7 ± 3.1	2.0 ± 0.9	-
CHI12	WP_026657636.1	G	D	E	N	K	R	S	45.9 ± 3.3	50.4 ± 2.8	28.8 ± 2.4	2.1 ± 0.3	-
CHI13	WP_095917646.1	G	D	E	R	T	R	S	14.5 ± 0.5	11.7 ± 0.4	2.2 ± 0.7	1.3 ± 0.3	-
CHI_{era}		G	N	D	K	Q	R	S	270.0 ± 9.4	90.7 ± 9.3	44.5 ± 0.8	31.5 ± 0.9	0.1 ± 0.0
CHI_{era}Mut1		G	R	E	N	K	G	S	32.6 ± 4.0	59.5 ± 2.4	23.7 ± 1.7	93.2 ± 1.5	0.4 ± 0.0
CHI_{era}Mut2		G	R	P	K	Q	G	S	4.4 ± 0.5	8.3 ± 0.2	3.5 ± 0.8	11.5 ± 0.3	-
CHI_{era}Mut3		G	R	E	K	N	G	S	16.7 ± 0.2	39.2 ± 1.0	13.0 ± 3.9	55.1 ± 4.9	0.3 ± 0.0
CHI_{era}Mut4		G	D	E	R	N	R	S	17.1 ± 4.0	52.4 ± 3.5	20.0 ± 10.3	30.3 ± 2.6	0.4 ± 0.0
CHI_{era}Mut5		G	D	E	R	T	R	S	61.4 ± 3.3	181.3 ± 6.0	62.2 ± 1.6	28.4 ± 0.6	-
CHI_{era}Mut6		G	E	P	N	K	R	S	25.6 ± 2.3	49.0 ± 5.0	21.2 ± 9.0	83.9 ± 5.4	0.5 ± 0.0
CHI_{era}Mut7		G	N	M	K	Q	R	S	67.5 ± 3.1	43.7 ± 1.3	14.4 ± 0.3	57.4 ± 3.8	-
CHI_{era}Mut8		G	D	I	K	Q	R	S	26.4 ± 4.8	60.0 ± 1.5	17.9 ± 2.1	96.4 ± 4.8	-
CHI_{era}Mut9		G	D	P	K	Q	R	S	76.8 ± 4.9	91.1 ± 7.1	30.8 ± 1.5	75.5 ± 4.8	-
CHI_{era}Mut10		A	R	P	N	K	R	S	11.0 ± 1.3	19.9 ± 0.5	9.8 ± 1.7	24.9 ± 0.5	-
CHI_{era}Mut11		D	D	P	N	K	R	S	28.0 ± 3.6	28.0 ± 0.9	33.6 ± 5.3	107.1 ± 9.7	1.4 ± 0.1
CHI_{era}Mut12		H	R	E	N	K	R	S	29.5 ± 1.8	27.2 ± 1.0	15.6 ± 1.0	4.5 ± 1.1	-

"-" means no activity observed. *The numbers refer to CHI_{era}.

Table S3. Primer list for mutagenesis of CHI_{era}

Primer name	Primer sequence
CHIera_R125G_f	AGATGCAGGAAATGCAGCCGGCAAATCCAACAATGCAGAAG
CHIera_R125G_b	CTTCTGCATTGTTGGATTTGCCGGCTGCATTTCCTGCATCT
CHIera_R125K_f	GGAGATGCAGGAAATGCAGCCAAAAAATCCAACAATGCAGAAGGA
CHIera_R125K_b	TCCTTCTGCATTGTTGGATTTTTTGGCTGCATTTCCTGCATCTCC
CHIera_R125G_G121A_f	GATGCAGCGAATGCAGCCGGCAAATCCAACAATGCAGAA
CHIera_R125G_G121A_b	TTCTGCATTGTTGGATTTGCCGGCTGCATTCGCTGCATC
CHIera_R125G_G121H_f	AGATGCACATAATGCAGCCGGCAAATCCAACAATGCAGAAG
CHIera_R125G_G121H_b	CTTCTGCATTGTTGGATTTGCCGGCTGCATTATGTGCATCT
CHIera_N122R_R125G_f	ATTCTGACGGAGATGCAGGACGCGCAGCCGGCAAATCCAAC
CHIera_N122R_R125G_b	GTTGGATTTGCCGGCTGCGCGTCCTGCATCTCCGTCAGAAT
CHIera_N122R_R125K_f	CGATTCTGACGGAGATGCAGGACGCGCAGCCAAAAAATCCAACAA
CHIera_N122R_R125K_b	TTGTTGGATTTTTTGGCTGCGCGTCCTGCATCTCCGTCAGAATCG
CHIera_N122R_G121A-R125G_f	CTGACGGAGATGCAGCGCGCGCAGCCGGCAAATCCAA
CHIera_N122R_G121A-R125G_b	TTGGATTTGCCGGCTGCGCGCGCTGCATCTCCGTCAG
CHIera_N122D_G121D_f	CTGACGGAGATGCAGATGATGCAGCCCGTAAATCC
CHIera_N122D_G121D_b	GGATTTACGGGCTGCATCATCTGCATCTCCGTCAG
CHIera_N122R_G121H-R125G_f	GATTCTGACGGAGATGCACATCGCGCAGCCGGCAAATCCAACA
CHIera_N122R_G121H-R125G_b	TGTTGGATTTGCCGGCTGCGCGATGTGCATCTCCGTCAGAATC
CHIera_N122E_f	CTGACGGAGATGCAGGAGAAGCAGCCCGTAAATCC
CHIera_N122E_b	GGATTTACGGGCTGCTTCTCCTGCATCTCCGTCAG
CHIera_G121A_f	GATTCTGACGGAGATGCAGCGAATGCAGCCCGTAAATCC
CHIera_G121A_b	GGATTTACGGGCTGCATTCGCTGCATCTCCGTCAGAATC
CHIera_G121D_f	GATTCTGACGGAGATGCAGATAATGCAGCCCGTAAATCC
CHIera_G121D_b	GGATTTACGGGCTGCATTATCTGCATCTCCGTCAGAATC
CHIera_G121H_f	ATCGATTCTGACGGAGATGCACATAATGCAGCCCGTAAATCCAAC
CHIera_G121H_b	GTTGGATTTACGGGCTGCATTATGTGCATCTCCGTCAGAATCGAT
CHIera_L87N_f	TCCTCGTCTTGAGATCAATGCAATCGCTGAGACAT
CHIera_L87N_b	ATGTCTCAGCGATTGCATTGATCTCAAGACGAGGA
CHIera_L87R_f	CTTGATCCTCGTCTTGAGATCCGTGCAATCGCTGAGACATTCCCG
CHIera_L87R_b	CGGGAATGTCTCAGCGATTGCACGGATCTCAAGACGAGGATCAAG
CHIera_D79I_f	GAGCATCACTGGTTAGTAAGCATTCTTGATCCTCGTCTTGAGATC
CHIera_D79I_b	GATCTCAAGACGAGGATCAAGAATGCTTACTAACCAGTGATGCTC
CHIera_D79P_f	GAGCATCACTGGTTAGTAAGCCCGCTTGATCCTCGTCTTGAGATC
CHIera_D79P_b	GATCTCAAGACGAGGATCAAGCGGGCTTACTAACCAGTGATGCTC
CHIera_D79M_f	GAGCATCACTGGTTAGTAAGCATGCTTGATCCTCGTCTTGAGATC
CHIera_D79M_b	GATCTCAAGACGAGGATCAAGCATGCTTACTAACCAGTGATGCTC
CHIera_D79E_f	TCACTGGTTAGTAAGCGAACTTGATCCTCGTCTTG
CHIera_D79E_b	CAAGACGAGGATCAAGTTCGCTTACTAACCAGTGA
CHIera_Q40K_f	TACATATTCCGGACAGCATCTCTAAGTTTGAGCCGT
CHIera_Q40K_b	ACGGCTCAAACTTAGAGATGCTGTCCGGAATATGTA
CHIera_Q40N_f	TATTCCGGACAGCATCTCTAACTTTGAGCCGTATGTTACCA
CHIera_Q40N_b	TGGTAACATACGGCTCAAAGTTAGAGATGCTGTCCGGAATA
CHIera_Q40T_f	GTACATATTCCGGACAGCATCTCTACCTTTGAGCCGTATGTTACCAAATAT
CHIera_Q40T_b	ATATTTGGTAACATACGGCTCAAAGGTAGAGATGCTGTCCGGAATATGTAC
CHIera_Q40I_f	AGTACATATTCCGGACAGCATCTCTATTTTTGAGCCGTATGTTACCAAATATG
CHIera_Q40I_b	CATATTTGGTAACATACGGCTCAAAAATAGAGATGCTGTCCGGAATATGTACT

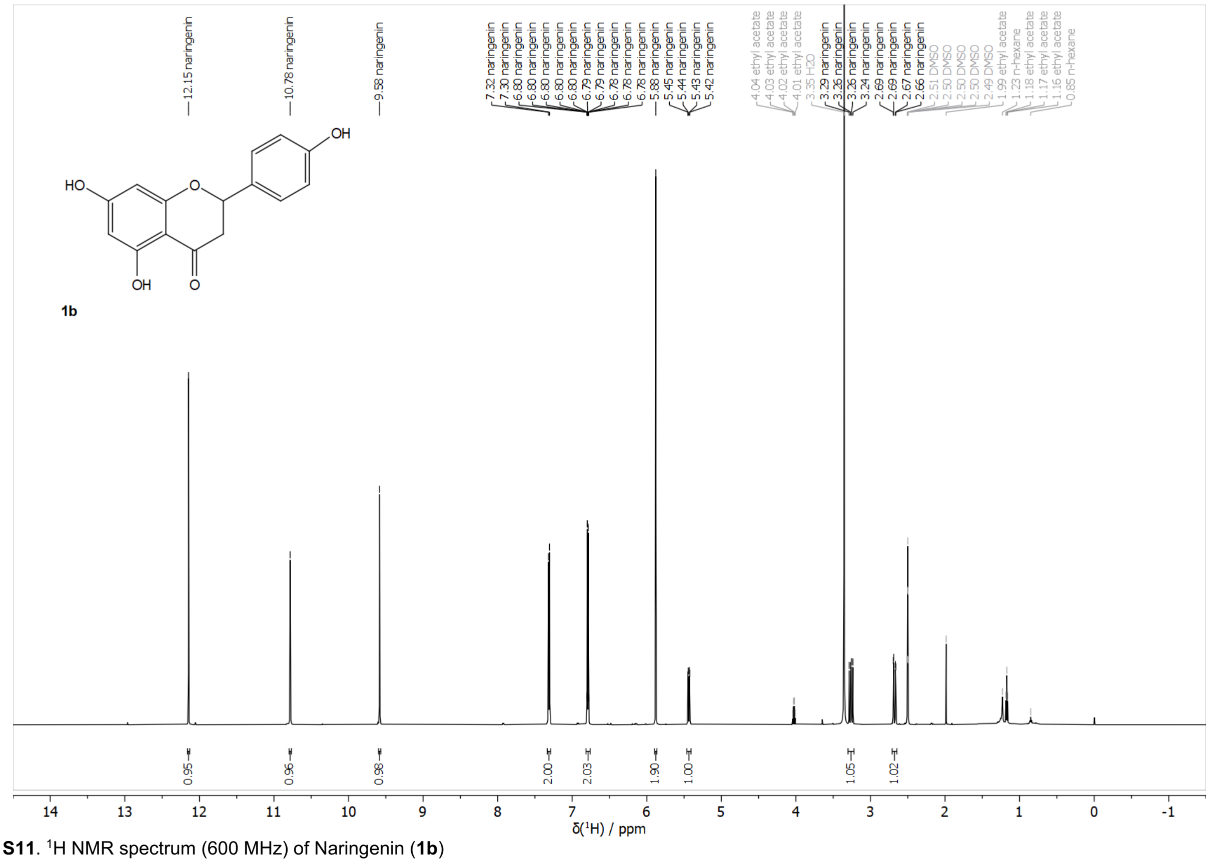

Figure S11. ^{1}H NMR spectrum (600 MHz) of Naringenin (**1b**)

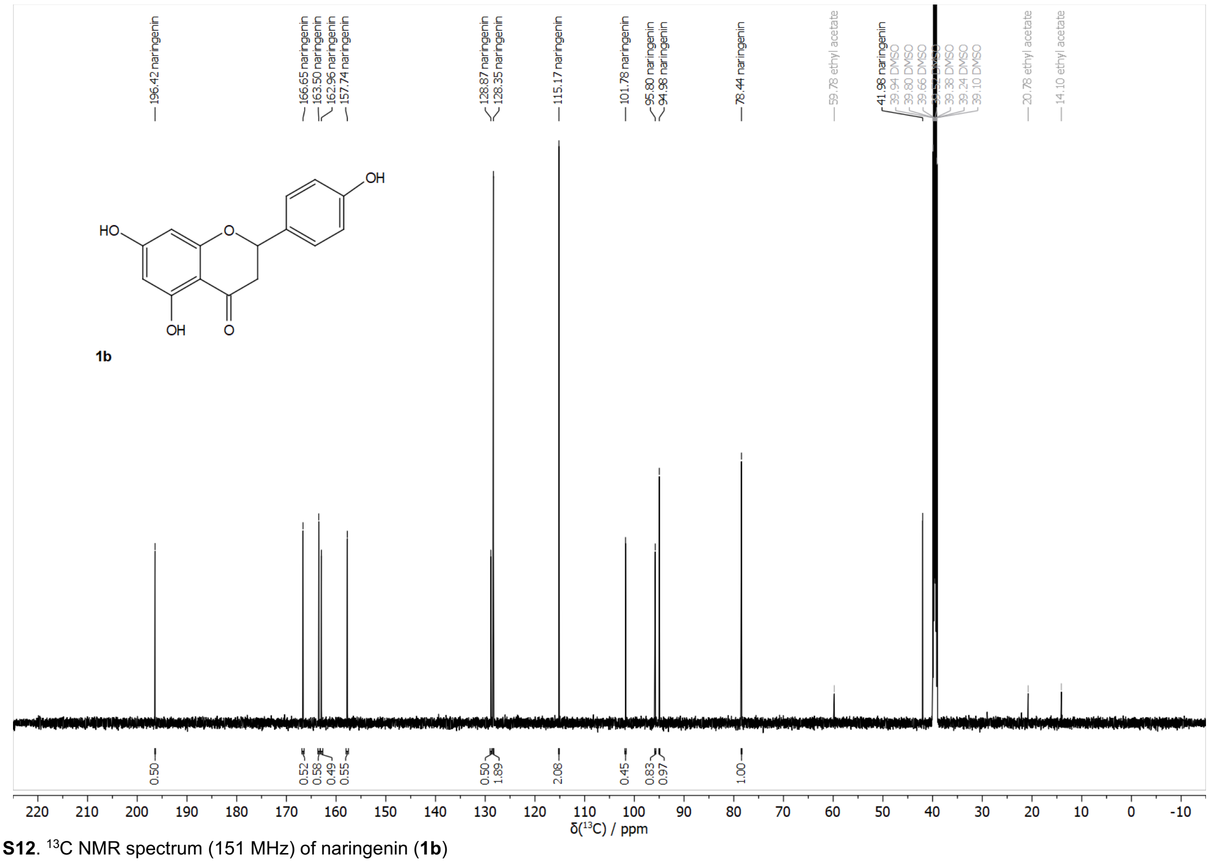

Figure S12. ^{13}C NMR spectrum (151 MHz) of naringenin (**1b**)

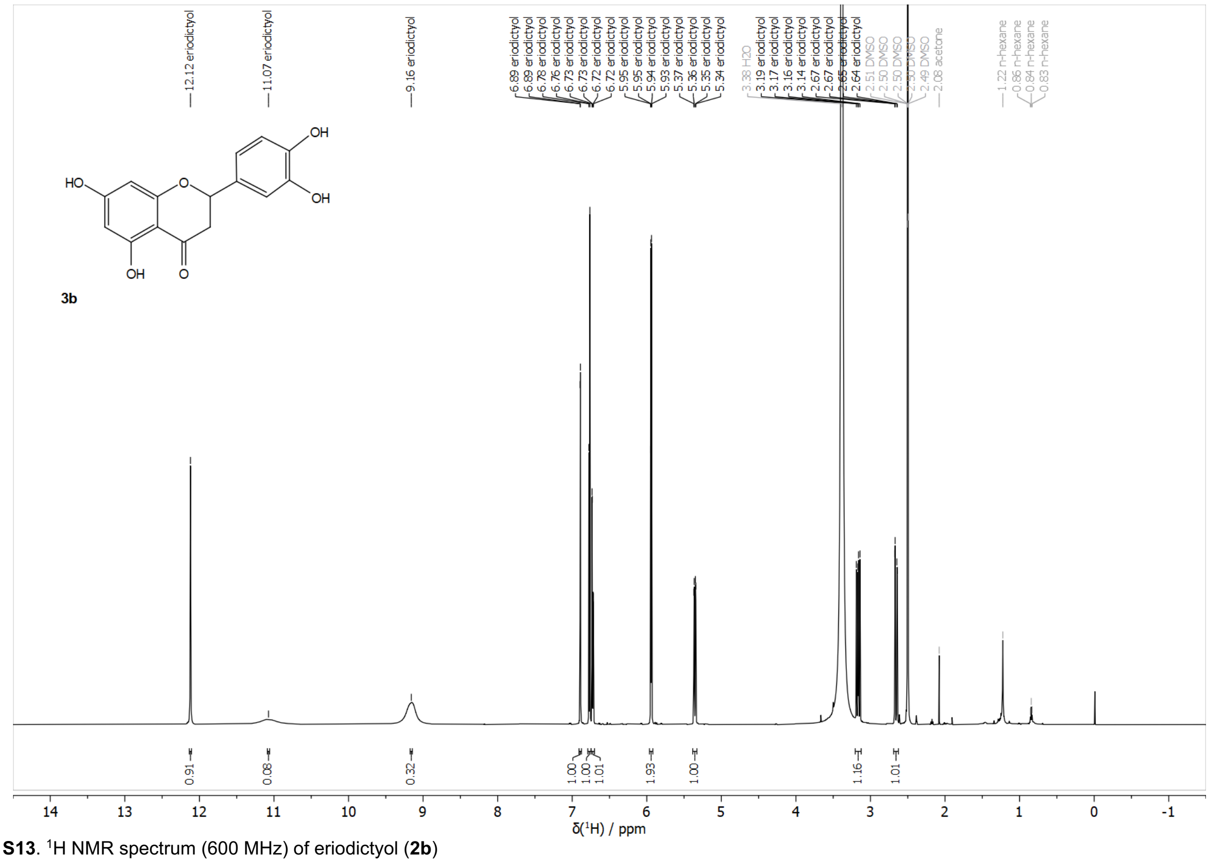

Figure S13. ^{1}H NMR spectrum (600 MHz) of eriodictyol (**2b**)

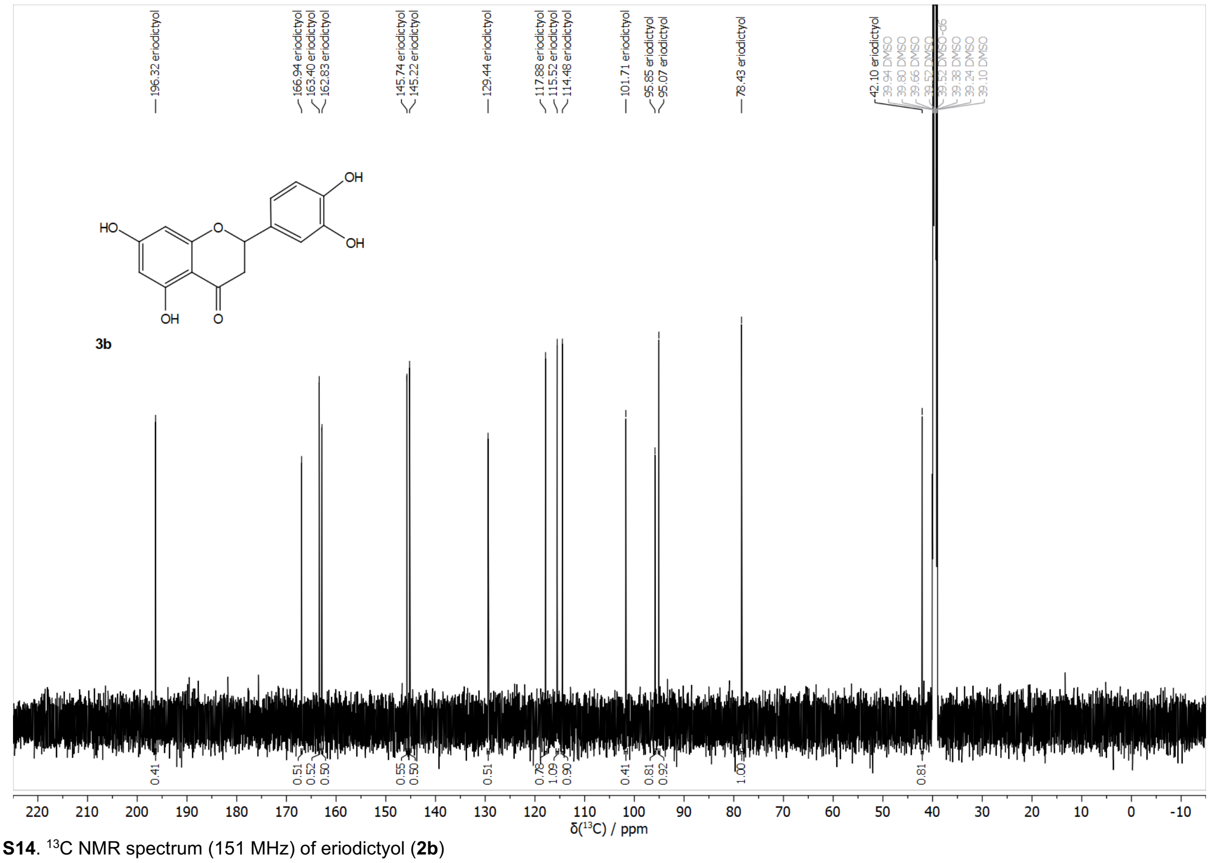

Figure S14. ^{13}C NMR spectrum (151 MHz) of eriodictyol (**2b**)

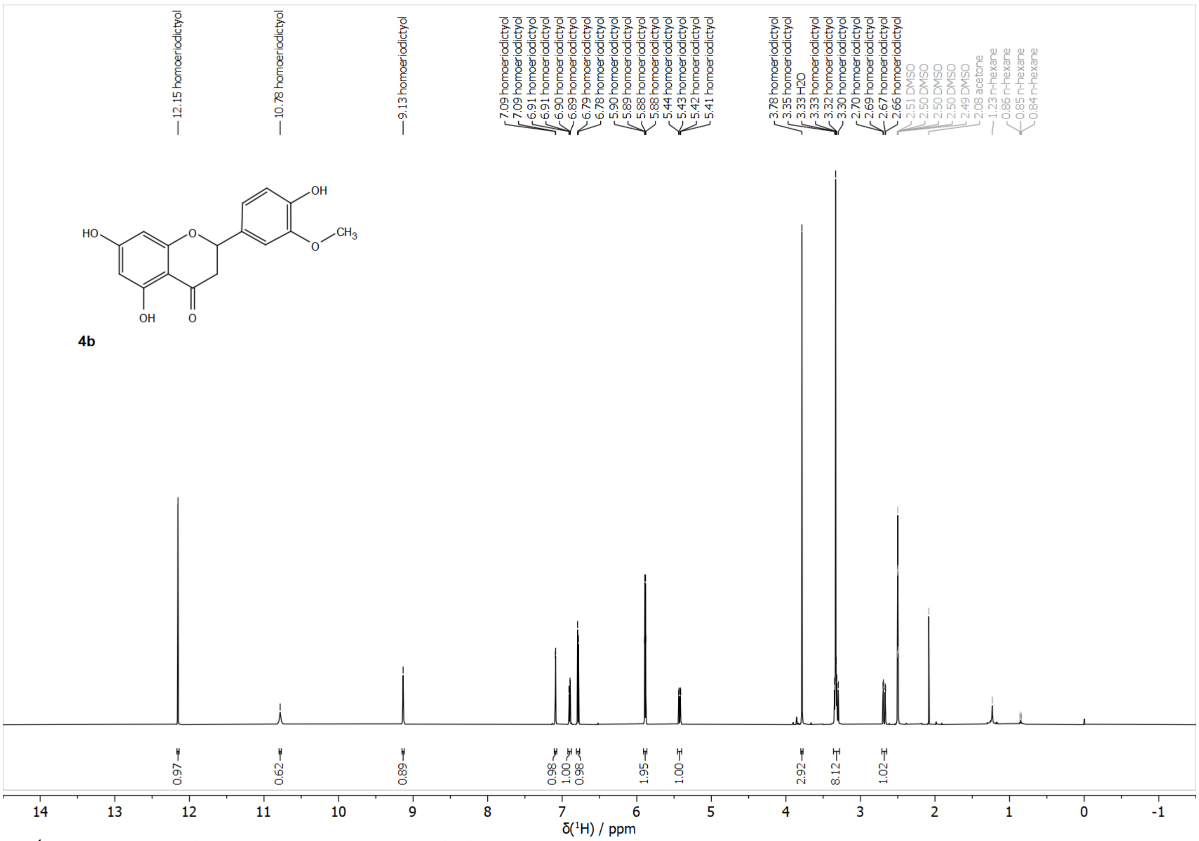

Figure S15. ^{1}H NMR spectrum (600 MHz) of homoeriodictyol (**3b**)

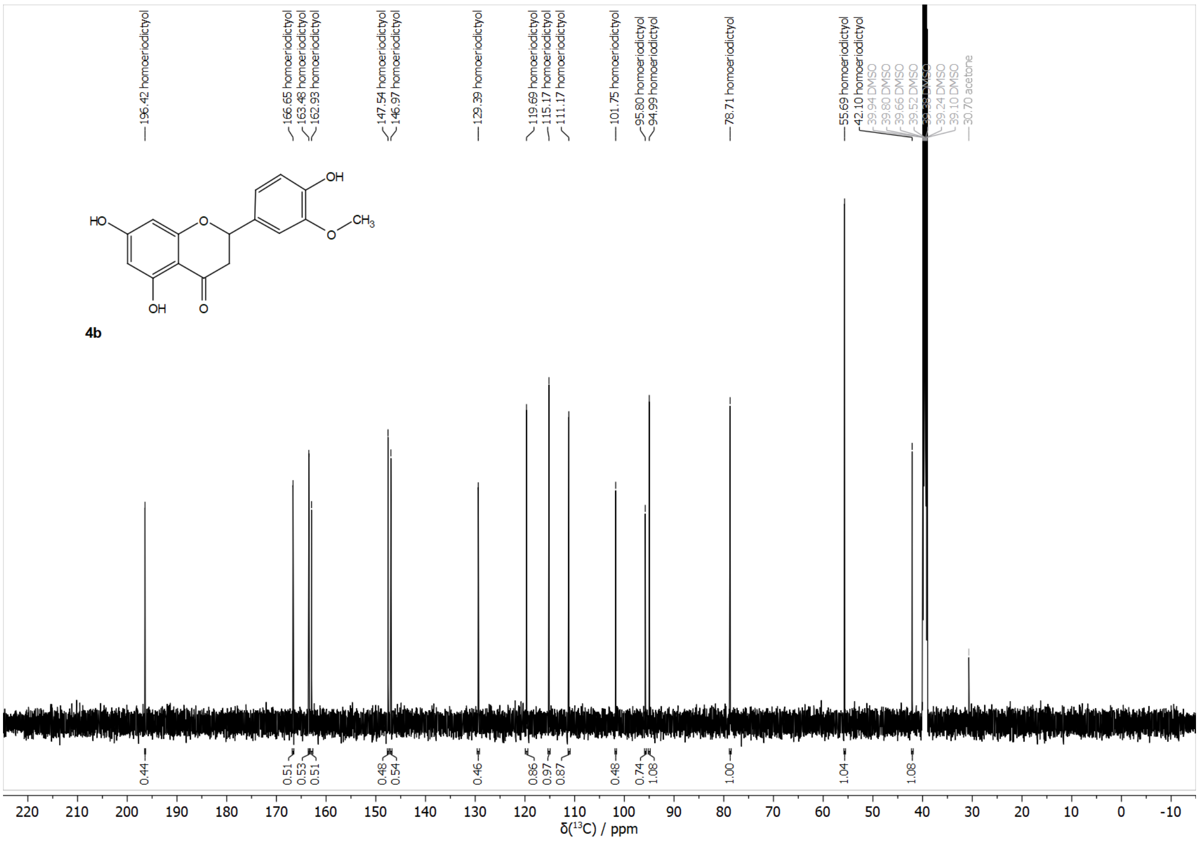

Figure S16. ^{13}C NMR spectrum (151 MHz) of homoeriodictyol (**3b**)

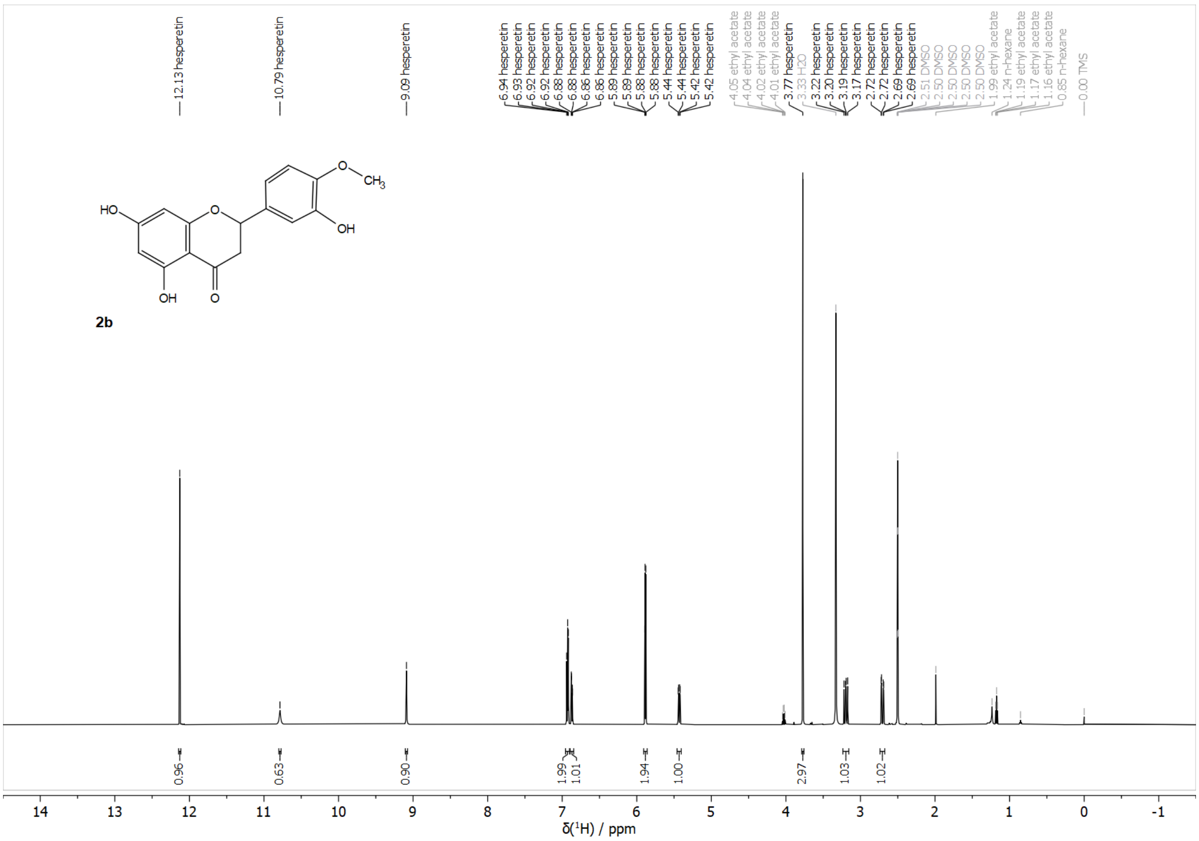

Figure S17. ^{1}H NMR spectrum (600 MHz) of hesperetin (**4b**)

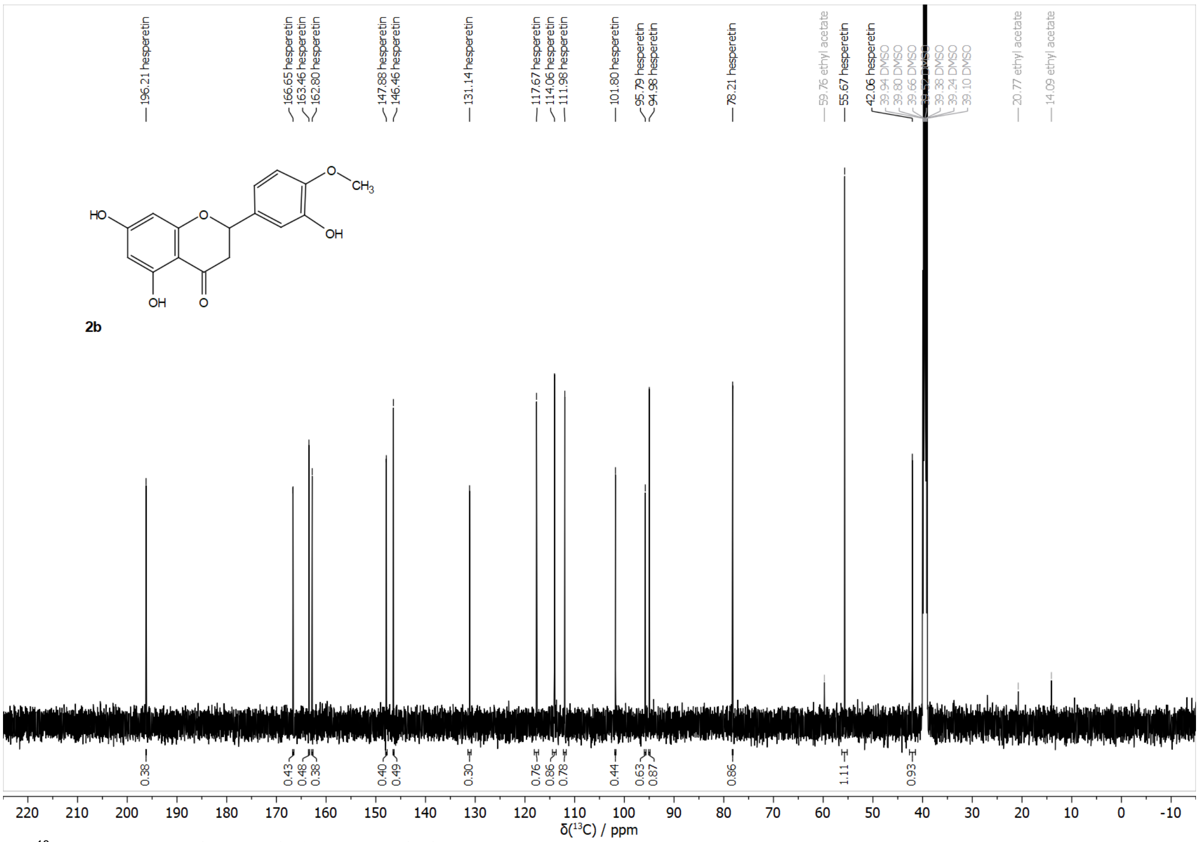

Figure S18. ^{13}C NMR spectrum (151 MHz) of hesperetin (**4b**)

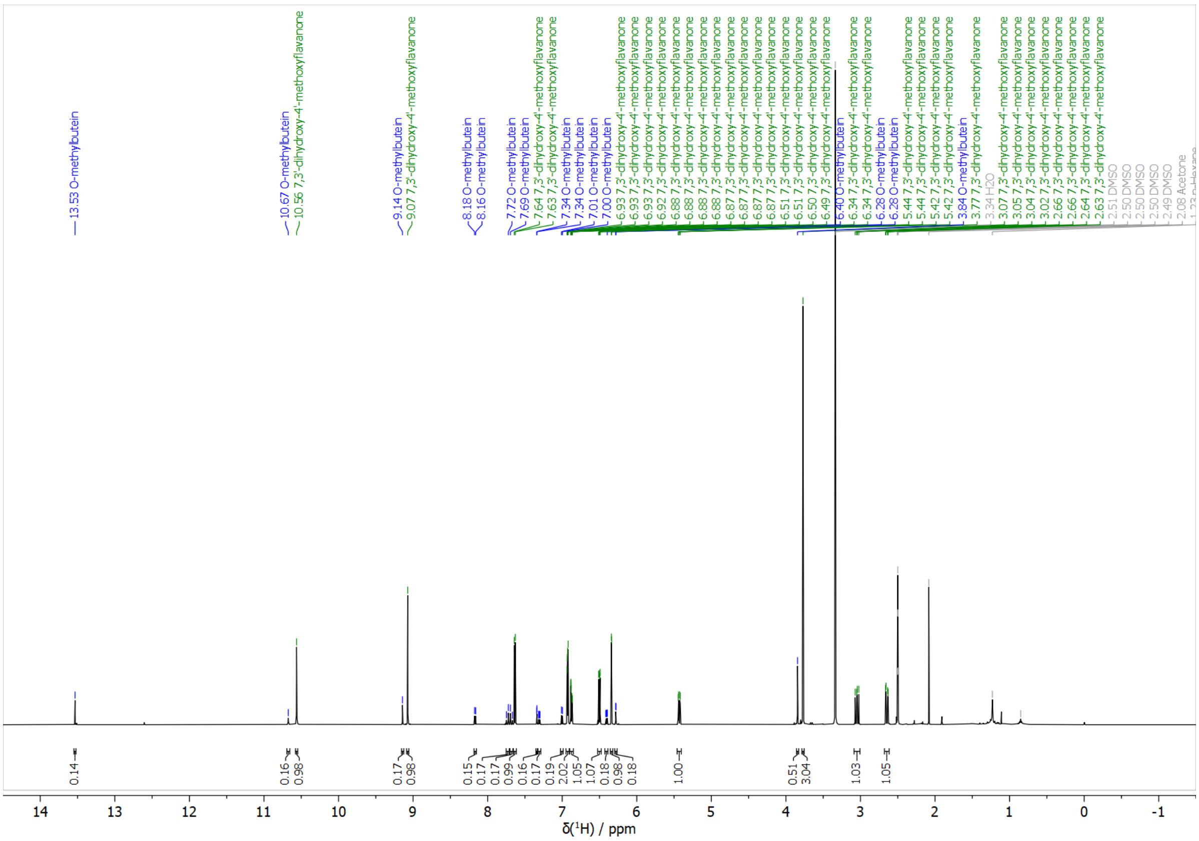

Figure S19. ^{1}H NMR spectrum (600 MHz) of 4-O-methylbutein (**5a**) and 7,3'-dihydroxy-4'-methoxyflavanone (**5b**)

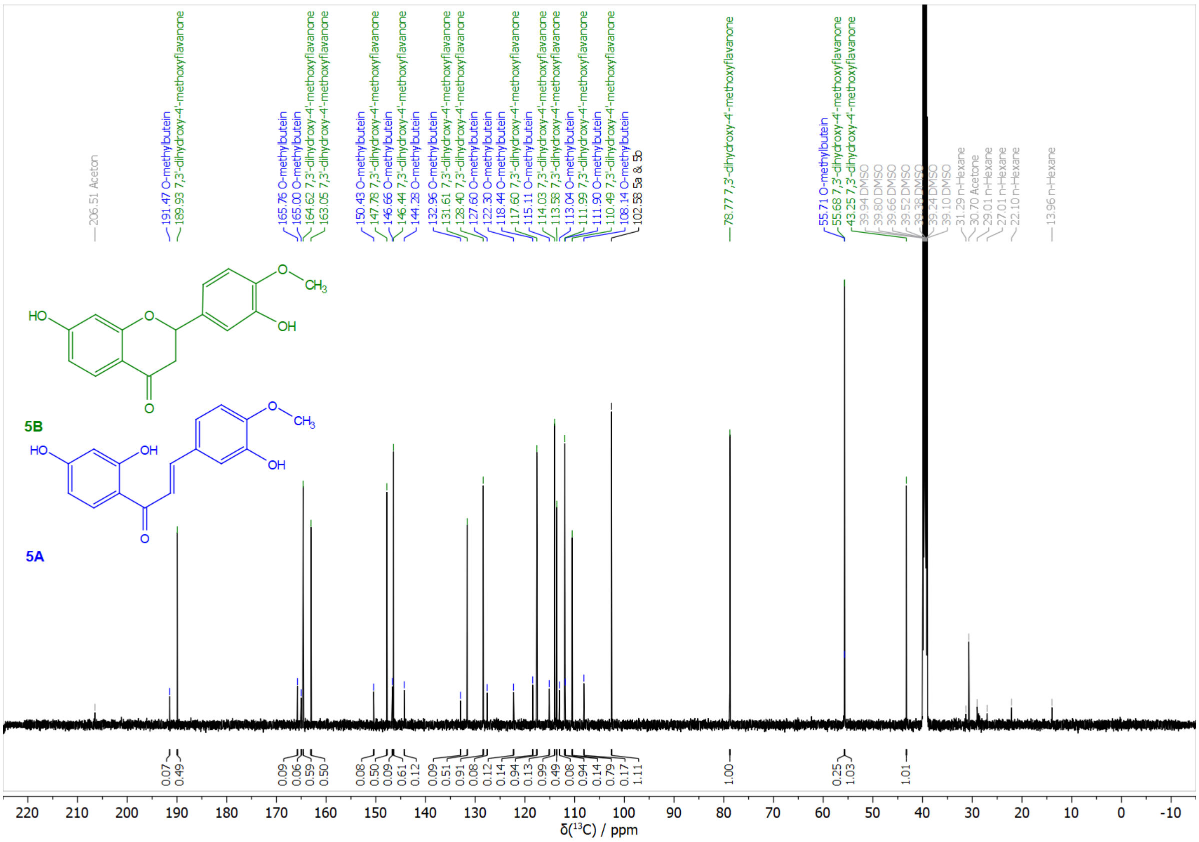

Figure S20. ^{13}C NMR spectrum (151 MHz) of 4-*O*-methylbutein (**5a**) and 7,3'-dihydroxy-4'-methoxyflavanone (**5b**)

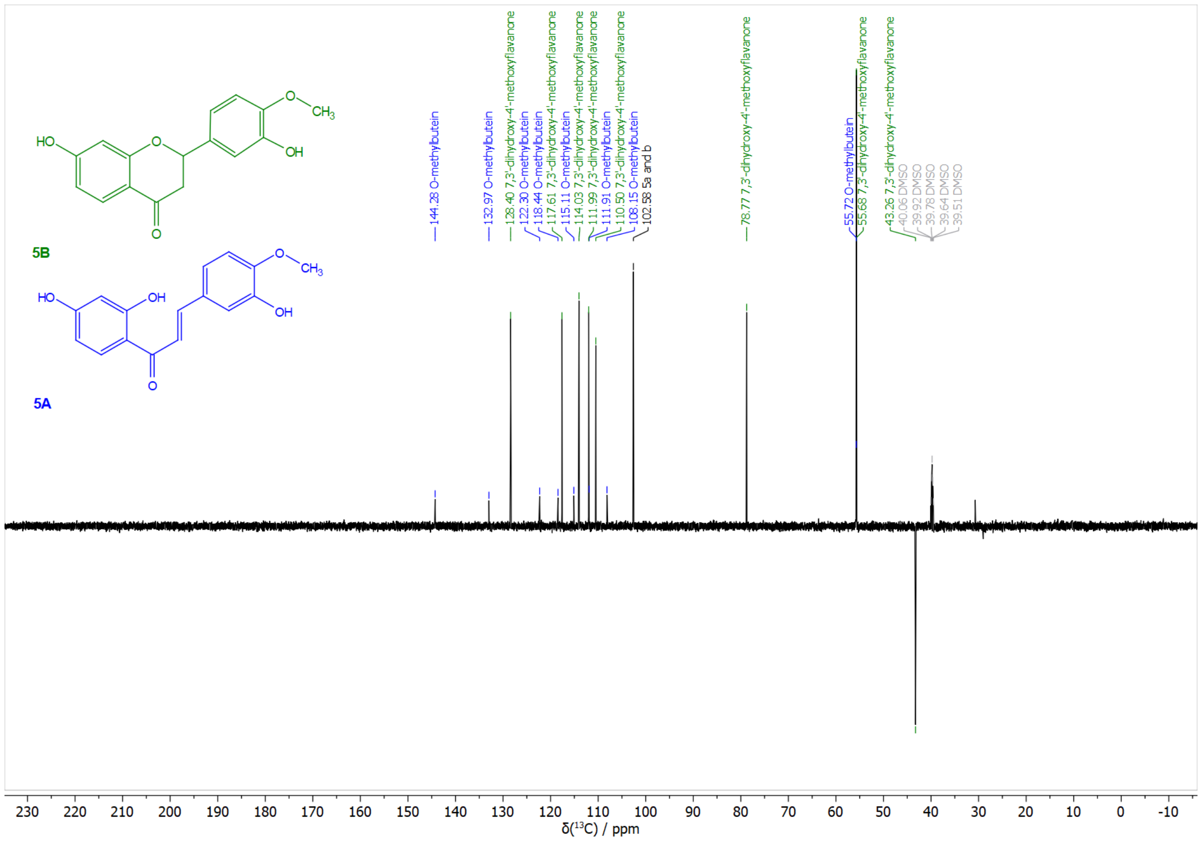

Figure S21. DEPT-135 NMR spectrum (151 MHz) of 4-O-methylbutein (**5a**) and 7,3'-dihydroxy-4'-methoxyflavanone (**5b**)

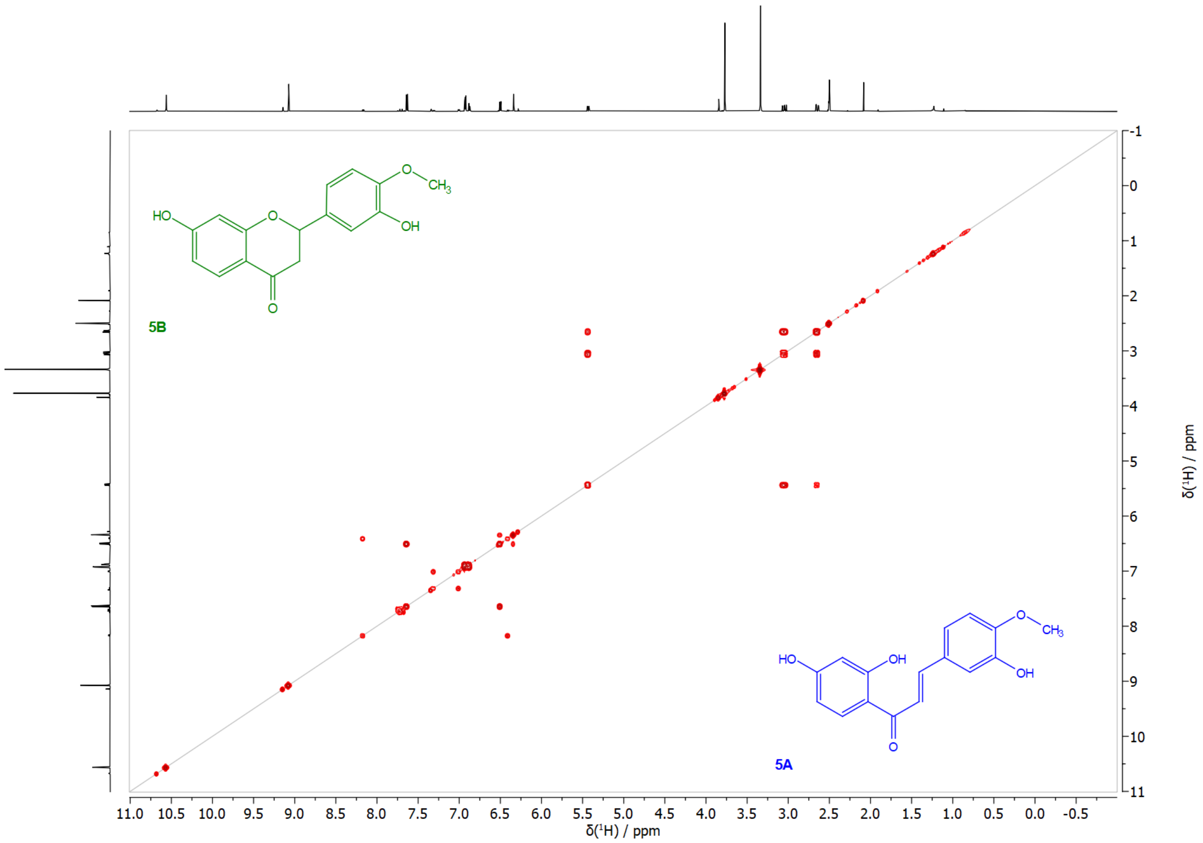

Figure S22. $^{1}H,^{1}H$-COSY NMR spectrum (600 MHz) of 4-*O*-methylbutein (**5a**) and 7,3'-dihydroxy-4'-methoxyflavanone (**5b**)

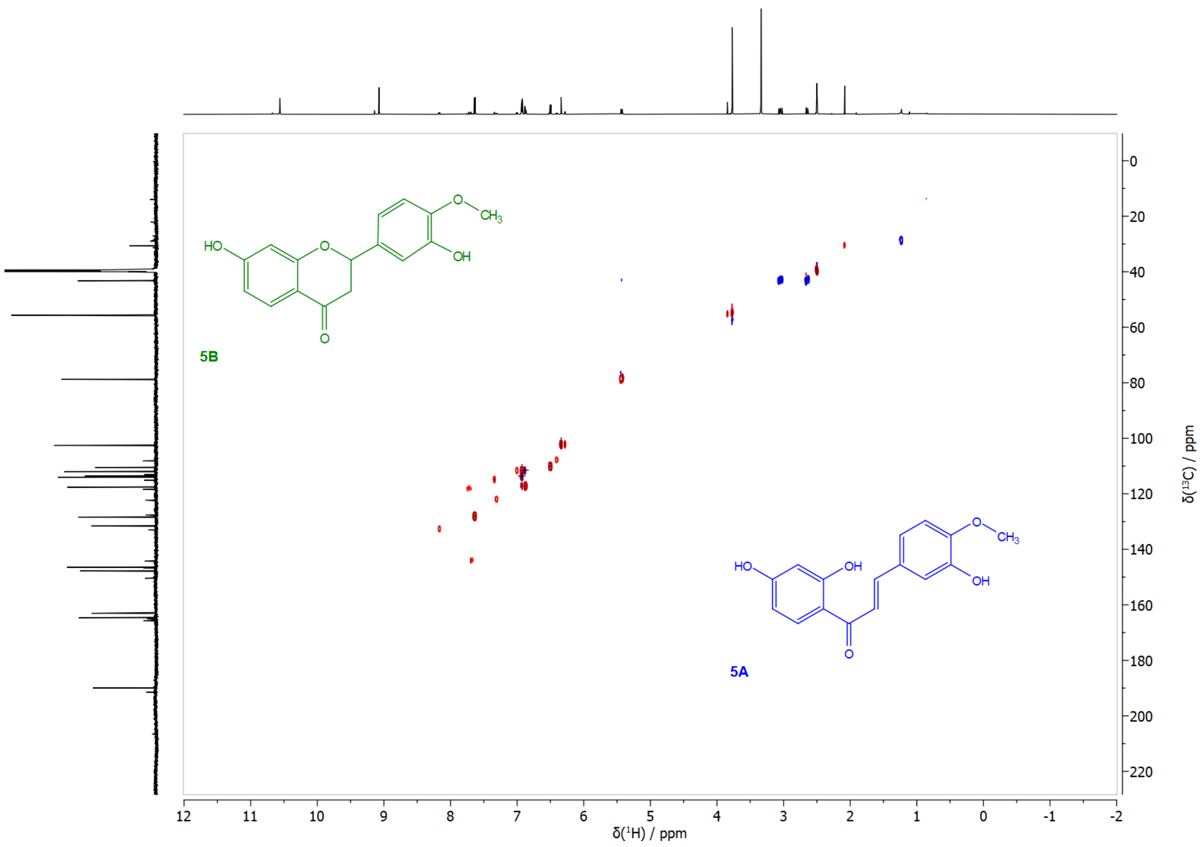

Figure S23. ^{1}H,^{13}C HSQC NMR spectrum (600 MHz / 151 MHz) of 4-*O*-methylbutein (**5a**) and 7,3'-dihydroxy-4'-methoxyflavanone (**5b**)

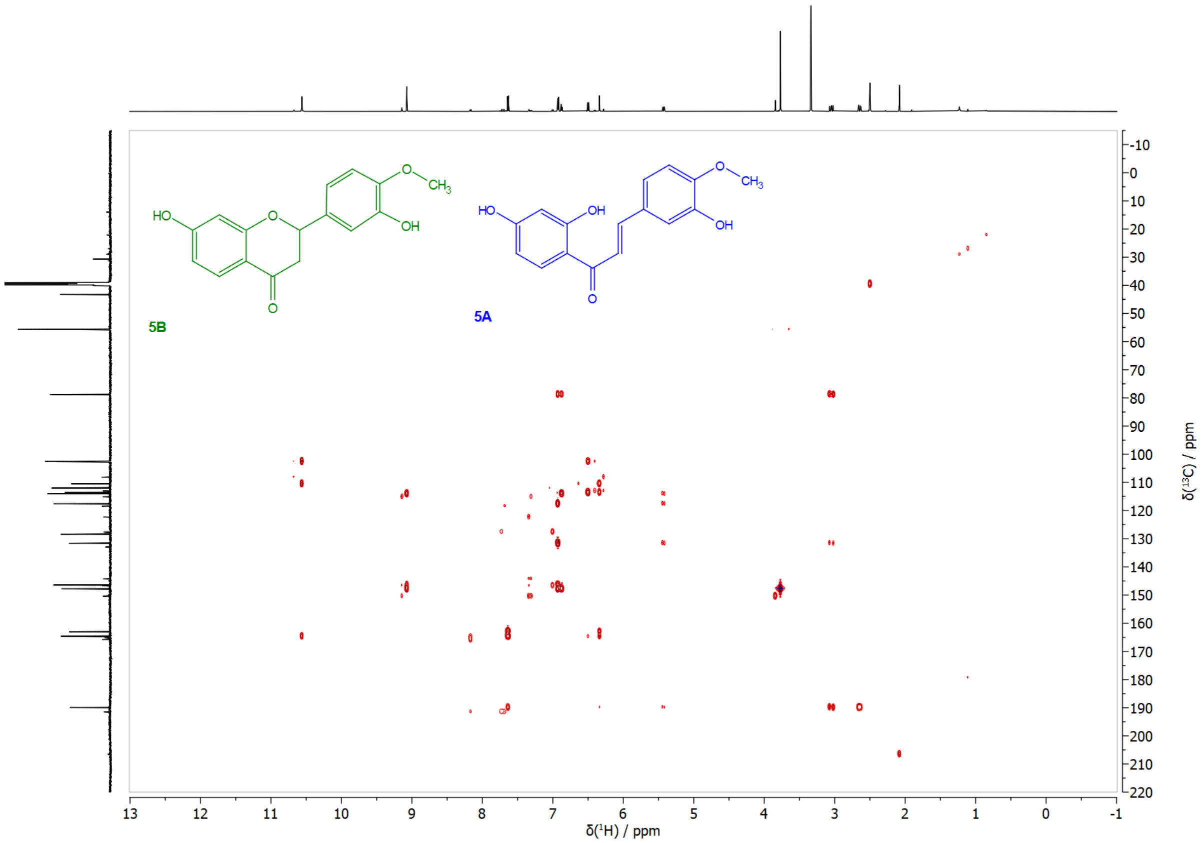

Figure S24. ^{1}H,^{13}C HMBC NMR spectrum (600 MHz / 151 MHz) of 4-*O*-methylbutein (5a) and 7,3'-dihydroxy-4'-methoxyflavanone (5b)

Structure elucidation details for 7,3'-dihydroxy-4'-methoxyflavanone

Table S4. Correlations identified via 2D-NMR experiments of 7,3'-dihydroxy-4'-methoxyflavanone

^{1}H,^{1}H COSY	^{1}H,^{13}C HMBC
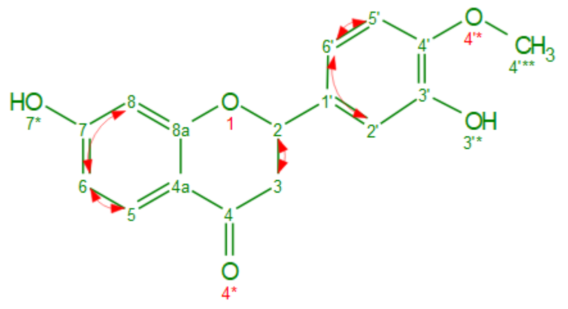	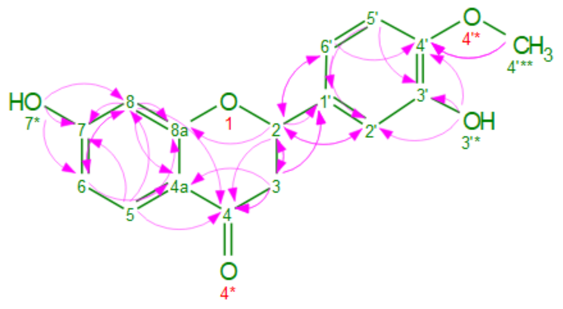

Table S5. Assignment details for 7,3'-dihydroxy-4'-methoxyflavanone

Atom	Nuclei	δ / ppm	COSY	HSQC	HMBC	J Hz (Atom)
**1'**	^{13}C	131.61			2, 3a, 3b, 5'	
**2**	^{13}C	78.77		2	2', 3a, 6'	
**2**	^{1}H	5.43	3a, 3b	2	1', 2', 3, 4, 6', 8a	3.04(3b), 12.48(3a)
**2'**	^{13}C	114.03		2'	2, 2', 3'*, 6'	
**2'**	^{1}H	6.93	6'	2'	2, 2'	2.15(6')
**3**	^{13}C	43.25		3a, 3b	2	
**3a**	^{1}H	3.05	2, 3b	3	1', 2, 4	12.48(2), 16.74(3b)
**3b**	^{1}H	2.65	2, 3a	3	1', 4, 4a	3.04(2), 16.74(3a)
**3'**	^{13}C	146.44			3'*, 5'	
**3'***	^{1}H	9.07			2', 3', 4'	
**4**	^{13}C	189.93			2, 3a, 3b, 5, 8	
**4'**	^{13}C	147.78			3'*, 4'**, 6'	
**4'****	^{13}C	55.68		4'**		
**4**	^{1}H	3.77		4'**	4'	
**4a**	^{13}C	113.58			3b, 6, 8	
**5**	^{13}C	128.40		5		
**4**	^{1}H	7.64	6	5	4, 7, 8, 8a	8.66(6)
**5'**	^{13}C	111.99		5'		
**5'**	^{1}H	6.93	6'	5'	1', 3'	8.27(6')
**6**	^{13}C	110.49		6	7*, 8	
**6**	^{1}H	6.50	5, 8	6	4a, 8	2.24(8), 8.66(5)
**6'**	^{13}C	117.60		6'	2	
**6'**	^{1}H	6.88	2', 5'	6'	2, 2', 4'	2.15(2'), 8.27(5')
**7**	^{13}C	164.62			5, 7*, 8	
**7**	^{1}H	10.56			6, 7, 8	
**8**	^{13}C	102.58		8	5, 6, 7*	
**8**	^{1}H	6.34	6	8	4, 4a, 6, 7, 8a	2.24(6)
**8a**	^{13}C	163.05			2, 5, 8	

Structure elucidation details for 4-*O*-methylbutein

Table S6. Correlations identified via 2D-NMR experiments of 4-*O*-methylbutein

^{1}H,^{1}H COSY	^{1}H,^{13}C HMBC
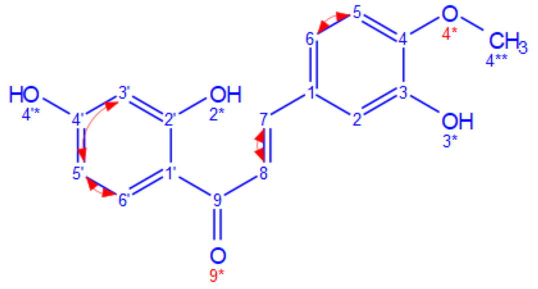	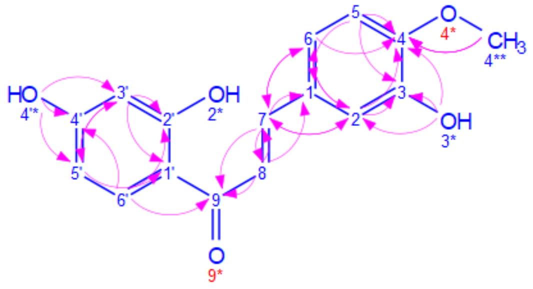

Table S7. Assignment details for 4-*O*-methylbutein

Atom	Nuclei	δ / ppm	COSY	HSQC	HMBC	J Hz (Atom)
**1**	^{13}C	127.60			5, 7, 8	
**1'**	^{13}C	113.04			3', 5'	
**2****	^{1}H	13.53				
**2**	^{13}C	115.11		2	3*, 6, 7	
**2**	^{1}H	7.34	2	2	3, 4, 6, 7	2.15(6)
**2'**	^{13}C	165.76			3', 6'	
**3**	^{13}C	146.66			2, 3*, 5	
**3***	^{1}H	9.14			2, 3, 4	
**3'**	^{13}C	102.58		3'	4'*, 5'	
**3'**	^{1}H	6.28	5'	3'	1', 2', 5'	2.37(5')
**4**	^{13}C	150.43				2, 3*, 4**, 5, 6
**4****	^{13}C	55.71		4**		
**4****	^{1}H	3.84		4**	4	
**4'**	^{13}C	165.00			4'*, 6'	
**4‘****	^{1}H	10.67			3', 4', 5'	
**5**	^{13}C	111.90		5		
**5**	^{1}H	7.00	6	5	1, 3, 4	8.36(6)
**5'**	^{13}C	108.14		5'	3', 4'*	
**5'**	^{1}H	6.41	3', 6'	5'	1', 3'	2.37(3'), 8.90(6')
**6**	^{13}C	122.30		6	2, 7	
**6**	^{1}H	7.31	5	6	2, 4, 7	8.36(5), 2.15(2)
**6'**	^{13}C	132.96		6'		
**6'**	^{1}H	8.17	5'	6'	2', 4', 9	8.90(5')
**7**	^{13}C	144.28		7	2, 6, 8	
**7**	^{1}H	7.68	8	7	1, 2, 6, 8, 9	15.25(8)
**8**	^{13}C	118.44		8	7	
**8**	^{1}H	7.73	7	8	1, 7, 9	15.25(7)
**9**	^{13}C	191.47			6', 7, 8	

Table S8. Results of qNMR experiments on flavanone and chalcone samples

#	Name	N_{Analyt}	δ_{Analyt} / ppm	Purity / %*
1b	naringenin	1	5.4	88.2 ± 7.1
2b	eriodictyol	1	5.4	37.8 ± 3.5
3b	homoeriodictyol	1	5.4	63.4 ± 5.2
4b	hesperetin	1	5.4	81.3 ± 8.2
5b	7,3'-dihydroxy-4'-methoxyflavanone	1	5.4	48.9 ± 3.8
5a	4-*O*-methylbutein	2	7.7	10.4 ± 2.3

*given via mean and confidence interval for α = 0.05

References

[1] M. Thomsen, A. Tuukkanen, J. Dickerhoff, G. J. Palm, H. Kratzat, D. I. Svergun, K. Weisz, U. T. Bornscheuer, W. Hinrichs, *Acta. Crystallogr. D Biol. Crystallogr.* **2015**, *71*, 907-917.
[2] Y. Huang, B. Niu, Y. Gao, L. Fu, W. Li, *Bioinformatics* **2010**, *26*, 680-682.
[3] C. Notredame, D. G. Higgins, J. Heringa, *J. Mol. Biol.* **2000**, *302*, 205-217.
[4] L. T. Nguyen, H. A. Schmidt, A. von Haeseler, B. Q. Minh, *Mol. Biol. Evol.* **2015**, *32*, 268-274.
[5] M. Gall, M. Thomsen, C. Peters, I. V. Pavlidis, P. Jonczyk, P. P. Grünert, S. Beutel, T. Scheper, E. Gross, M. Backes, T. Geißler, J. P. Ley, J. M. Hilmer, G. Krammer, G. J. Palm, W. Hinrichs, U. T. Bornscheuer, *Angew. Chem. Int. Ed.* **2014**, *53*, 1439-1442; *Angew. Chem.* **2014**, *126*, 1463-1466.
[6] M. K. Khan, N. Rakotomanomana, C. Dufour, O. Dangles, *Food Funct.* **2011**, *2*, 617-626.

Author Contributions

U.T.B. and J.P.L. conceived the project and designed the experiments together with D.Y., T.G. and B.Z.; H.M. and D.Y. performed most experiments supported by B.Z. and E.S.; B.H. performed the NMR measurements and evaluated the results. S.B. also evaluated the NMR results and wrote the supplementary part for the NMR results. C.R. performed the chiral measurements and analytics. E.G. performed original experiments on chiral distribution with native CHI. D.Y. drafted the manuscript, which was revised by all authors.
